# c-MYC-dependent transcriptional inhibition of autophagy is implicated in cisplatin sensitivity in HPV-positive head and neck cancer

**DOI:** 10.1038/s41419-023-06248-3

**Published:** 2023-11-04

**Authors:** Alessandro Medda, Micaela Compagnoni, Giorgio Spini, Simona Citro, Ottavio Croci, Stefano Campaner, Marta Tagliabue, Mohssen Ansarin, Susanna Chiocca

**Affiliations:** 1 Department of Experimental Oncology, IEO, European Institute of Oncology IRCCS, IEO Campus, Via Adamello 16, 20139 Milan, Italy; 2 Center for Genomic Science of IIT, CGS@SEMM (Istituto Italiano di Tecnologia at European School of Molecular Medicine), Fondazione Istituto Italiano di Tecnologia (IIT), Milan, Italy; 3 Division of Otolaryngology Head & Neck Surgery, IEO, European Institute of Oncology IRCCS, 20141 Milan, Italy; 4 Department of Biomedical Sciences, University of Sassari, Sassari, Italy

**Keywords:** Oral cancer, Macroautophagy, Oncogenes

## Abstract

Autophagy is important for the removal, degradation and recycling of damaged organelles, proteins, and lipids through the degradative action of lysosomes. In addition to its catabolic function, autophagy is important in cancer and viral-mediated tumorigenesis, including Human Papillomavirus (HPV) positive cancers. HPV infection is a major risk factor in a subset of head and neck cancer (HNC), for which no targeted therapies are currently available. Herein, we assessed autophagy function in HPV-positive HNC. We showed that HPV-positive HNC cells presented a transcriptional and functional impairment of the autophagic process compared to HPV-negative cells, which were reactivated by knocking down HPV E6/E7 oncoproteins, the drivers of cellular transformation. We found that the oncoprotein c-MYC was stabilized and triggered in HPV-positive cell lines. This resulted in the reduced binding of the MiT/TFE transcription factors to their autophagy targets due to c-MYC competition. Thus, the knock-down of c-MYC induced the upregulation of autophagic and lysosomal genes in HPV-positive HNC cells, as well as the increase of autophagic markers at the protein level. Moreover, HPV oncoprotein E7 upregulated the expression of the phosphatase inhibitor CIP2A, accounting for c-MYC upregulation and stability in HPV+ HNC cells. CIP2A mRNA expression negatively correlated with autophagy gene expression in tumor tissues from HNC patients, showing, for the first time, its implication in a transcriptional autophagic context. Both CIP2A and c-MYC knock-down, as well as pharmacological downregulation of c-MYC, resulted in increased resistance to cisplatin treatment. Our results not only show a novel way by which HPV oncoproteins manipulate the host machinery but also provide more insights into the role of autophagy in chemoresistance, with possible implications for targeted HPV-positive HNC therapy.

## Introduction

Head and neck cancer (HNC) comprises tumors originating in the epithelial cells of the mucosa of the upper aerial tract, including the oropharynx, the oral cavity, the larynx and the hypopharynx [1]. Based on its main risk factors, alcohol/tobacco consumption and Human Papillomavirus (HPV) infections, HNC can be subdivided into two subgroups: HPV-negative (HPV−) and HPV-positive (HPV+) [2]. HPV-related HNC mostly occurs in the oropharyngeal subsite and has been increasing in the last decades, especially in men [3]. Indeed, HPV+ oropharyngeal cancers have one of the fastest rises in incidence in non-Hispanic white men in the United States [4]. Although the two HNC subtypes are different in both clinical and molecular aspects, they are still treated with the same therapies, including surgery, when possible, followed by radio- and chemotherapy [5]. A better knowledge of the molecular aspects that distinguish these two subgroups would be beneficial for the discovery of novel targets and biomarkers for targeted therapy.

HPV is a sexually transmitted double-strand DNA virus of approximately 8000 bp [6]. Over 200 HPV strains exist, subdivided into high-risk or low-risk for the capability to induce carcinogenesis [7]. The main drivers of oncogenesis are the E6 and E7 oncoproteins: E6 interacts with many cellular proteins, including p53, which, upon interaction with the human ubiquitin-ligase E6AP, is sent to proteasomal degradation, leading to inhibition of apoptosis [8]. E7 mainly induces retinoblastoma protein (pRb) inactivation, leading to E2F1 release, an important transcription factor involved in cell cycle regulation, resulting in increased proliferation [9, 10].

Another target gene activated by E2F1 is the Cellular Inhibitor of PP2A (Protein Phosphatase 2A) (CIP2A) [11, 12]. CIP2A sequesters PP2A B56 regulatory subunit inhibiting its activity and, as a consequence, increases the half-life of the oncogene c-MYC by inhibiting its S62 dephosphorylation by PP2A [13].

Autophagy is a self-consumption intracellular process important for catabolism and homeostasis [14]. Macroautophagy, hereafter referred to as autophagy, consists in the formation of a double membrane organelle, the autophagosome [15], on which the light chain protein 3 (LC3) is loaded upon maturation to a lipidated form through a ubiquitin-like system [16, 17]. LC3 is extensively used to monitor the autophagic flux [18]. The autophagosome, containing a cargo of proteins and damaged organelles, is degraded upon fusion with lysosomes, degradative organelles consisting of digestive enzymes and an acidic environment [19]. Autophagy is important for the recycling of amino acids, the degradation of long-lived proteins, and the recycling of damaged organelles, including mitochondria [20]. Autophagy and lysosomal biogenesis can be transcriptionally regulated by different transcription factors, in particular, the microphthalmia/transcription factor E (MiT/TFE) family [21, 22]. This family includes microphthalmia-associated transcription factor (MITF), transcription factor E3 (TFE3), transcription factor EB (TFEB) and transcription factor EC (TFEC) proteins, belonging to the larger family of basic helix-loop-helix leucine zipper (bHLH-Zip) transcription factors (that also include c-MYC), that can recognize a specific motif (GTCACGTGAC) known as the coordinated lysosomal expression and regulation (CLEAR) [22]. Specifically, TFEB and TFE3 activities are controlled by phosphorylation: mechanistic target of rapamycin complex 1 (mTORC1) inhibition dephosphorylates TFEB/TFE3 inducing their nuclearization and activation, leading to the activation of lysosome/autophagy gene expression [23].

The role of autophagy in cancer is still not well understood. Many works suggest that autophagy inhibition drives tumorigenesis [24–26]; however, other studies suggest that autophagy induction can ensure cancer cell survival in advanced solid tumors [27, 28]. Autophagy is considered a double-edged sword in cancer [29].

The role of HPV in autophagy regulation is emerging, and in particular, E6 and E7 oncoproteins impinge this pathway at different steps [30]. E6 has been shown to inhibit late steps of autophagy in primary keratinocytes [31], while E7 has been observed to inhibit autophagy by AMBRA1 degradation [32].

In this study, we focused on autophagy gene expression regulation mediated by E6/E7 in HNC and how c-MYC affects this process. We also tested the implication of PP2A activation or c-MYC downregulation in combination with cisplatin in HPV+ HNC to assess their role in chemosensitivity.

## Results

### Autophagy is structurally and functionally impaired in HPV+ HNC cell lines

**Fig. 1:**
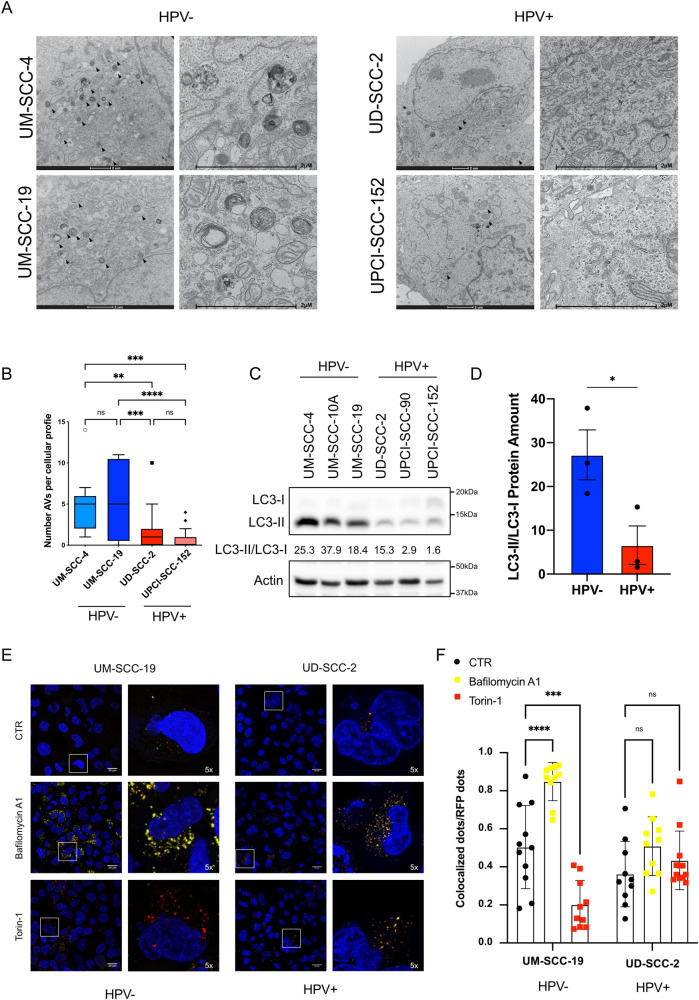
Structural and functional characterization of autophagy in HNC cell lines. **A** Representative Electron Micrographs showing subcellular compartments in two HPV− (UM-SCC-4 and UM-SCC-19) and two HPV+ (UD-SCC-2 and UPCI-SCC-152) cell lines. The panels on the right represent a magnification of the panels on the left. Arrows indicate autophagic vacuoles (AVs). **B** Boxplots show the number of AVs per cellular profile (*n* > 19). Box are expressed as means, including Tukey analysis. Statistical analysis was performed using ordinary one-way ANOVA corrected comparing False Discovery Rate (two-stage step-up method of Benjamini, Krieger and Yakuteli). **C** Western blot shows LC3 proteins levels in three HPV− (UM-SCC-4, UM-SCC-10A and UM-SCC-19) and three HPV+ (UD-SCC-2, UPCI-SCC-90, UPCI-SCC-152) HNC cell lines. Actin was used as loading control. LC3-II/LC3-I ratio is reported. **D** Histogram shows the densitometric protein levels of LC3-II/LC3-I ratio from figure 1C ± SEM. Unpaired *t*-test was performed. **E** Confocal micrographs of HPV− (UM-SCC-19) and HPV+ (UD-SCC-2) cell lines transduced with GFP-RFP-LC3 (Red and Green dots) and treated with 10 nM Bafilomycin A1, 1 µM Torin-1 or vehicle (CTR) for 24 h. The panels on the right represent 5x zoom of boxes on the left panels. Nuclei were stained with DAPI (Blue). **F** Quantification of colocalized dots for each RFP dot. Each dot represents a different field. Statistical analysis was performed using two-way ANOVA test with Dunnett’s correction (*n* > 9). Bars are expressed as mean ± SD.

To assess the differences in autophagy activation between HPV+ and HPV− HNC cells, we examined the number of autophagic vacuoles (AVs) (which include autophagosomes, autolysosomes and amphisomes) by electron microscopy (EM) in 2 HPV− and 2 HPV+ HNC cell lines (Fig. 1A). HPV+ HNC cell lines showed a reduced number and size of AVs compared to HPV− ones, suggesting a possible inhibition of the pathway (Fig. 1B, Supplementary Fig. S1A).

We then assessed the levels of autophagy markers in HNC cell lines at basal conditions. HPV+ HNC cell lines showed lower levels of the lipidated form of LC3 (LC3-II) compared to HPV− HNC cell lines (Fig. 1C, D), while other autophagy markers like Beclin or p62 were only respectively slightly or not affected (Supplementary Fig. S1B, C). We also measured the activation of Akt and mTORC, described to inhibit autophagy [33], by detecting Akt and p70 S6 kinase (S6K) phosphorylation by western blot in HNC cell lines (Supplementary Fig. S1D, E). While Akt phosphorylation at S473 seems not to be related to HPV status, p70 (S6K) phosphorylation can be detected in the UD-SCC-2 and UPCI-SCC-90 HPV+ HNC cell lines, suggesting that mTORC activity could promote autophagy inhibition in some HPV+ HNC cell lines.

To functionally characterize autophagy status, we monitored the autophagic flux by using a tandem tagged LC3-GFP-RFP fusion protein [34]. GFP is quenched in acidic pH such that only red dots can be observed in functional autolysosomes, while in neutral pH red and green colocalized dots can be detected. The HPV− UM-SCC-19 and the HPV+ UD-SCC-2 HNC cell lines transduced with the lentiviral LC3-GFP-RFP vector were treated with the late-stage autophagic inhibitor Bafilomycin A1, the mTORC1 inhibitor Torin-1, or the vehicle (DMSO) and observed by confocal microscopy (Fig. 1E). Quantification of colocalized GFP and RFP dots showed a significant increase upon autophagy inhibition and a significant decrease upon autophagy activation with respect to the vehicle only in the HPV− HNC cell lines, as expected in cells in which autophagy is functional (Fig. 1F, left). However, the levels of colocalized dots in the HPV+ HNC cell lines do not vary significantly with respect to the vehicle, upon pharmacological induction or inhibition of autophagy (Fig. 1F, right). Taken together, these results suggest a global impairment in autophagic functions in HPV+ HNC cell lines.

### HPV16 oncoproteins transcriptionally inhibit autophagy gene expression

**Fig. 2:**
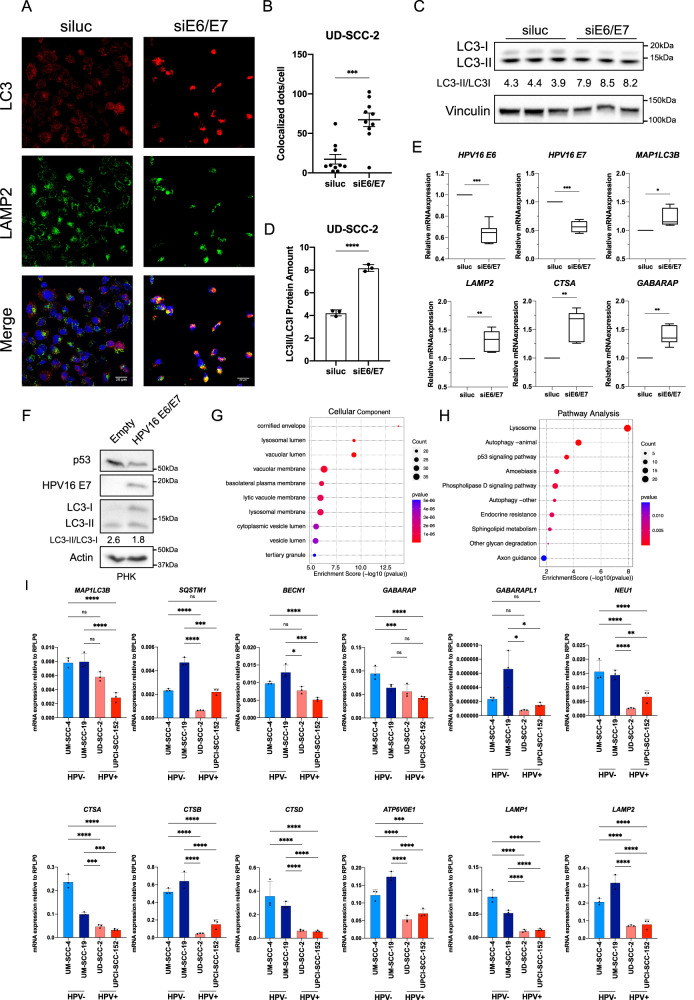
Effects of HPV E6/E7 on autophagy and autophagy gene expression. **A** IF staining of LC3 (red) and LAMP2 (green) in UD-SCC-2 cells transfected with siE6/E7 or the control siRNA (siluc). Nuclei were stained with DAPI (Blue). **B** Quantification of Colocalized LAMP2 and LC3 dots/cell in UD-SCC-2 transfected with siE6/E7 or the control siRNA (siluc). Unpaired *t*-test was performed, bars represent mean ± SEM (*n* = 10). **C** Western blot showing LC3 protein levels in three biological replicates of UD-SCC-2 cells upon transfection with siE6/E7 or siluc. Vinculin was used as loading control. LC3-II/LC3-I ratio is reported. **D** Histogram shows the densitometric protein levels of LC3-II/LC3-I. Unpaired *t*-test was performed. Bars represent mean ± SD (*n* = 3). **E** Boxplots showing mRNA expression of UD-SCC-2 cells transfected with siE6/E7 compared to the siluc. Boxes express min to max. Statistical analysis was performed using *t*-test (*n* = 6). **F** Western blot shows LC3 protein levels in PHK transduced with HPV16 E6/E7 or the empty vector. HPV16E7 and p53 were used as control of E7 and E6 respectively. Actin was used as loading control. LC3-II/LC3-I ratio is reported. **G** GO (Gene Ontology) enrichment bubble plot of genes downregulated by E6/E7 transduction in PHK, Cellular Compartment (*n* = 4). **H** KEGG pathway enrichment bubble plot of genes downregulated by E6/E7 transduction in PHK (*n* = 4). **I** Histograms show RT-qPCR results of UM-SCC-4, UM-SCC-19, UD-SCC-2 and UPCI-SCC-152 cell lines, representing mRNA expression of autophagy and lysosomal genes relative to the housekeeping gene (RPLP0) using 2^(-dCT) method. Statistical analysis was performed using ordinary one-way ANOVA with Bonferroni correction. Bars represent mean ± SD (*n* = 3).

To test whether HPV16 oncoproteins are involved in autophagy inhibition in HPV+ HNC cells, we knocked down E6/E7 by RNAi in UD-SCC-2 cells. We then stained Lysosome-associated membrane protein 2 (LAMP2) (lysosomal) and LC3 (autophagosomal) proteins, revealing an increase in colocalization upon reduction of E6/E7, suggesting a reactivation of the pathway (Fig. 2A, B). This result was confirmed also by a significant increase in the LC3II/LC3I ratio upon siE6/E7 (Fig. 2C, D). Interestingly, the downregulation of E6 and E7 was able to induce the mRNA upregulation of some autophagic and lysosomal genes (Fig. 2E).

To investigate the role of E6 and E7 in mRNA regulation of autophagy and lysosomal biogenesis, we transduced primary human keratinocytes (PHK) with HPV16 E6 and E7 (Fig. 2F, Supplementary Fig. S2A), followed by RNA sequencing (Supplementary Fig. S2D, E). Gene ontology analysis on genes downregulated by E6/E7 indicated genes belonging to lysosomal and vacuolar cellular compartments as some of the most relevant genes affected by the two oncoproteins (Fig. 2G), in addition, the pathway analysis highlighted lysosome and autophagy pathway among the top regulated hits (Fig. 2H). Moreover, HPV16 E6 and E7 alone, seem to induce mRNA downregulation of autophagy and lysosomal biogenesis (Supplementary Fig. S2E–G). These results were also confirmed by qPCR analysis (Supplementary Fig. S2B, C).

Next, we wanted to confirm these results also in HNC cell lines by monitoring the mRNA expression of autophagy and lysosomal genes in two HPV− (UM-SCC-4, UM-SCC-19) and two HPV+ (UD-SCC-2, UPCI-SCC-152) HNC cell lines at basal conditions. Figure 2I shows that many autophagy (the *LC3* gene *MAP1LC3B*, the p62 gene *SQSTM1*, *BECN1*, *GABARAP*, *GABARAPL1*) and, more strongly, lysosomal (*NEU1*, *CTSA*, *CTSB*, *CTSD*, *ATP6V0E1*, *LAMP1*, *LAMP2*) genes are differentially expressed between HPV− and HPV+ cell lines. These data suggest that HPV16 oncoproteins transcriptionally downregulate autophagy and lysosomal biogenesis.

### c-MYC competes with TFEB and TFE3 for the binding to autophagy and lysosomal genes in HPV+ HNC cells

**Fig. 3:**
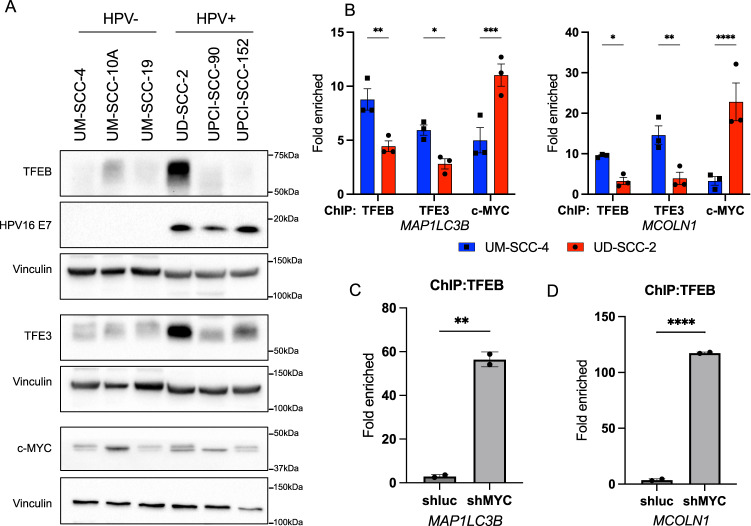
c-MYC, TFEB and TFE3 binding to autophagy gene promoters in HNC cell lines. **A** Western blots showing TFEB, TFE3 and c-MYC protein levels in HNC cell lines. HPV16 E7 is the control for HPV+ cell lines, Vinculin is used as loading control. **B** Histograms represent TFEB (*n* = 3), TFE3 (*n* = 3), and c-MYC (*n* = 3) binding to the promoters of *MAP1LC3B* and *MCOLN1*, assessed by ChIP qPCR, and expressed as fold enrichment to the IgG in UD-SCC-2 (HPV+) or UM-SCC-4 (HPV-) cell lines. Statistical analysis was performed using two-way ANOVA corrected comparing False Discovery Rate (two-stage step-up method of Benjamini, Krieger and Yakuteli). Bars express mean ± SD. **C** Histogram represents TFEB binding to the promoter of *MAP1LC3B*, assessed by ChIP qPCR, and expressed as fold enrichment to the IgG in UD-SCC-2 transduced with shMYC or shluc. Statistical analysis was performed using *t*-test (*n* = 2). Bars express mean ± SD. **D** Histogram represents TFEB binding to the promoter of *MCOLN1*, assessed by ChIP qPCR, and expressed as fold enrichment to the IgG in UD-SCC-2 transduced with shMYC or shluc. Statistical analysis was performed using *t*-test (*n* = 2). Bars express mean ± SD.

As transcriptional repression of autophagy and lysosomal biogenesis occurred in HPV+ cells, we investigated the molecular mechanism underlying this process. We evaluated the mRNA and protein expression (Supplementary Fig. S3A–C, Fig. 3A) of the most important transcription factors involved in lysosomal biogenesis: TFEB and TFE3, as well as c-MYC, recently found to repress autophagy [35]. Western blots performed on HNC cell lines did not show any significant difference in the expression of these genes between HPV− and HPV+ cell lines (Fig. 3A).

Thus, we focused on the differential activation of the transcription factors, and on their binding to the promoters of autophagy genes. We performed chromatin immunoprecipitation (ChIP) of TFEB, TFE3, and c-MYC in the UM-SCC-4 and UD-SCC-2 cell lines, followed by qPCR analysis, using primers targeting the promoters of autophagy and lysosomal genes in a region containing the TFs consensus motif (see supplementary information section). We found that the binding of c-MYC to the promoter of LC3 gene (*MAP1LC3B*) and *MCOLN1* were significantly higher in the HPV+ cell line (UD-SCC-2) compared to the HPV− cell line (UM-SCC-4), and this corresponded to a significant decrease in the binding of TFEB and TFE3 to the same promoter (Fig. 3B). The same result was partially confirmed also for *GLA* and *ATP6V1H* promoters (Supplementary Fig. S3D), while no significant differences could be appreciated for the promoter of the p62 gene (*SQSTM1*) (Supplementary Fig. S3D). These observations are in line with Annunziata et al. [35] study, which showed that c-MYC competes with TFE3 and TFEB, repressing lysosomal and autophagic transcription. To confirm the competition in HPV+ HNC cells, we knocked down c-MYC and performed ChIP of TFEB in UD-SCC-2 cells. The reduction of c-MYC strikingly increased the binding of TFEB to the promoter of different autophagy and lysosomal genes (Fig. 3C, D, Supplementary Fig. S3E–G). Our data suggest that HPV+ HNC cells present a more active c-MYC protein compared to HPV− cells, which prevents the binding of TFEB and TFE3 to autophagy and lysosomal promoters.

### c-MYC stability increases in HPV+ HNC cells, affecting the autophagy pathway

**Fig. 4:**
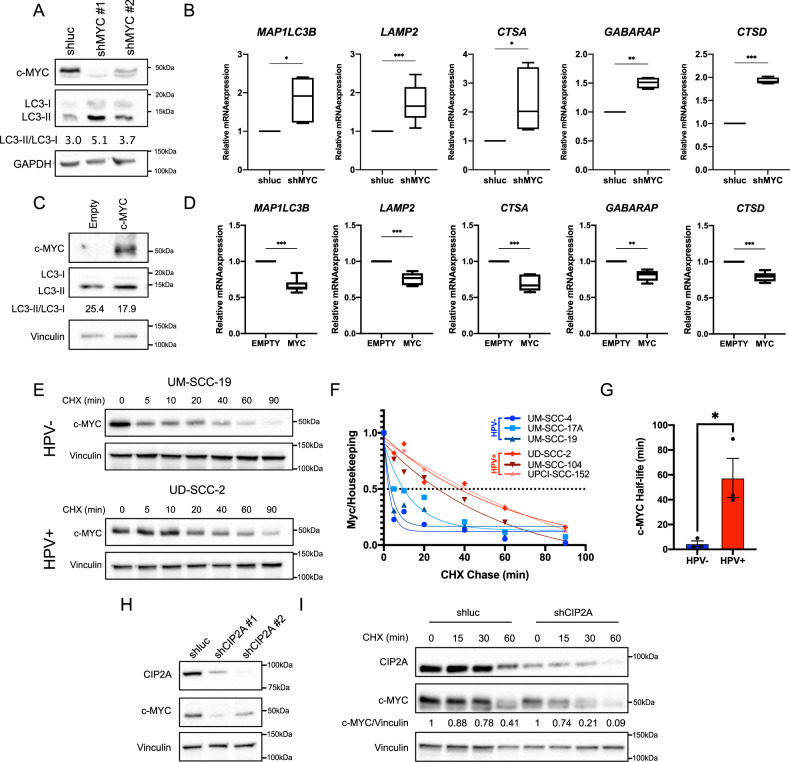
c-MYC is stabilized and inhibits autophagy in HPV+ HNC cells. **A** Western blot showing LC3 regulation upon knock-down of c-MYC in UPCI-SCC-152 cells. Vinculin is used as loading control. LC3-II/LC3-I ratio is reported. **B** Boxplots showing mRNA expression of autophagy genes in UPCI-SCC-152 cells transduced with shMYC compared to the shluc. Whiskers express Tukey analysis. Statistical analysis was performed using *t*-test (*n* > 4). **C** Western blot showing LC3 regulation upon overexpression of c-MYC in UD-SCC-2 cells. Vinculin is used as loading control. LC3-II/LC3-I ratio is reported. **D** Boxplots showing mRNA expression of autophagy genes in UD-SCC-2 cells overexpressing c-MYC compared to the empty vector. Whiskers express Tukey analysis. Statistical analysis was performed using *t*-test (*n* = 6). **E** Western blots show c-MYC protein levels at different time points (expressed in minutes) after cycloheximide treatment (CHX) (UM-SCC-19 and UD-SCC-2 cell lines). Vinculin is used as loading control. **F** Graph representing c-MYC protein quantification (normalized to the loading control) upon CHX Chase over time (expressed in minutes) in three HPV− (UM-SCC-4, UM-SCC-17A, UM-SCC-19) and three HPV+ (UD-SCC-2, UPCI-SCC-90, UPCI-SCC-152) HNC cell lines. **G** Histogram showing c-MYC half-life in HPV− and HPV+ cell lines. Statistical analysis was performed using *t*-test. Bars represent mean ± SD. **H** Western blot shows c-MYC protein levels upon knock-down of CIP2A in UD-SCC-2 cells. **I** Western blot showing c-MYC protein levels at different time points (expressed in minutes) after cycloheximide treatment (CHX) in UD-SCC-2 cells transduced with shCIP2A or shluc. Vinculin is used as housekeeping. c-MYC/ Vinculin protein ratio is reported.

To assess whether c-MYC is involved in the inhibition of autophagy, we performed c-MYC knock-down in HPV+ HNC cell lines. c-MYC downregulation affected both protein expression of LC3 and the mRNA levels of different autophagy genes (Fig. 4A, B, Supplementary Fig. S4A, B, D). Moreover, c-MYC overexpression in UD-SCC-2 and UPCI-SCC-152 cell lines reduced LC3-II/LC3-I protein ratio and induced the downregulation of autophagy genes, confirming the involvement of c-MYC in autophagy repression (Fig. 4C,D, Supplementary Fig. S4C).

Since no significant difference in the mRNA expression of *MYC* between HPV+ and HPV− HNC cell lines was detected (Supplementary Fig. S3C), we evaluated the stability of c-MYC protein by treating HNC cell lines with cycloheximide (CHX), a widely used inhibitor of protein synthesis, at different time points. Since c-MYC is very unstable, we used 5 min as the first time point and 90 min as the last. Western blot analysis revealed a higher stability of c-MYC in HPV+ with respect to HPV− HNC cell lines (Fig. 4E, Supplementary Fig. S4E, F). We calculated the half-life of c-MYC in 3 HPV− and 3 HPV+ HNC cell lines (Fig. 4F) and we observed a significantly higher half-life in the HPV+ HNC cell lines, revealing a higher stability of c-MYC in these cell lines (Fig. 4G).

Since c-MYC stability is affected by its phosphorylation, we tried to assess whether the phosphatase inhibitor CIP2A was responsible for c-MYC stabilization in the UD-SCC-2 cell line. Silencing of CIP2A by shRNA induced a strong downregulation of c-MYC protein levels (Fig. 4H), which was specifically imputable to the reduction of c-MYC half-life (Fig. 4I). These results confirmed our hypothesis that, also in our system, c-MYC is regulated by CIP2A expression.

### CIP2A inhibits autophagy and lysosomal biogenesis in HNC

**Fig. 5:**
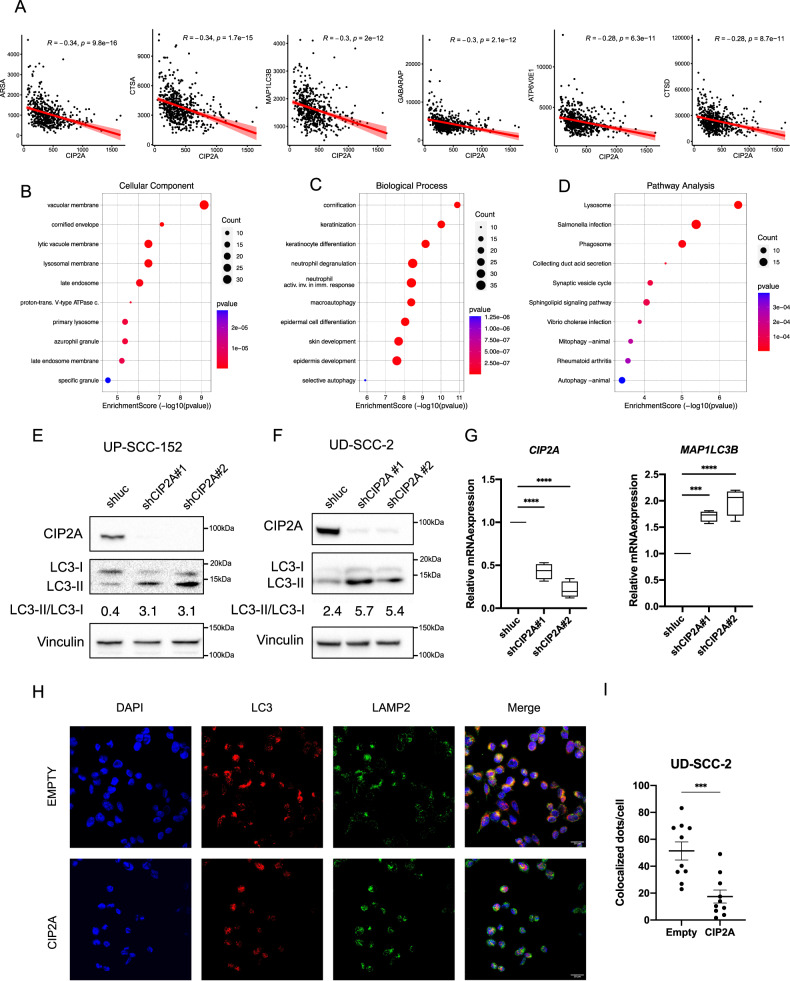
CIP2A expression affects autophagy in HNC. **A** Scatter plots express the Pearson correlation (red line) between mRNA expression of CIP2A and autophagy/lysosomal genes (RSEM) in HNC samples within the Pancancer dataset from the TCGA. **B**–**D** GO and KEGG enrichment bubble plots of top 100 genes inversely correlated with CIP2A expression in HNC samples within the Pancancer dataset from the TCGA: Cellular compartment (**B**), Biological process (**C**) and KEGG pathway (**D**). **E**, **F** Western blot shows LC3 protein levels upon knock-down of CIP2A in UPCI-SCC-152 (**E**) and UD-SCC-2 (**F**) cell lines. Vinculin was used as loading control. LC3-II/LC3-I ratio is reported. **G** Boxplots showing mRNA expression of MAP1LC3B or CIP2A in UD-SCC-2 cells transduced with shCIP2A compared to the shluc. Whiskers express Tukey analysis. Statistical analysis was performed using *t*-test (*n* = 4). **H** IF staining of LC3 (red) and LAMP2 (green) in UD-SCC-2 cells transfected with CIP2A or the empty vector. Nuclei were stained with DAPI (Blue). **I** Quantification of Colocalized LAMP2 and LC3 dots/cell in UD-SCC-2 transfected with CIP2A or the empty vector. Unpaired *t*-test was performed. Bars represent mean ± SD (*n* = 10).

As CIP2A affected c-MYC stability, we investigated the role of *CIP2A* mRNA expression in HNC tumors. We took advantage of the Pancancer Dataset of Head and Neck cancer tumors from the Cancer Genome Atlas (TCGA) and assessed the correlation between CIP2A mRNA and autophagy or lysosomal gene expression. We found that many genes involved in autophagy and lysosomal biogenesis inversely correlated with CIP2A expression (Fig. 5A, Supplementary Fig. S5A–C). We then took the top 100 genes that inversely correlated with *CIP2A* expression and performed a gene ontology analysis, looking at cellular components, biological processes and pathways enriched (Fig. 5B–D). As shown in Fig. 5B–D, among the most enriched hits we found lysosome-related genes, macroautophagy, mitophagy and selective autophagy.

We then decided to assess whether CIP2A expression affects autophagy in HPV+ HNC cell lines. Upon CIP2A silencing in HPV+ HNC cell lines we showed the upregulation of LC3-II (Fig. 5E, F), and also an increased *MAP1LC3B* gene expression (Fig. 5G). Moreover, the overexpression of CIP2A reduced the colocalization of LC3 with LAMP2 (Fig. 5H, I). Taken together these results evidence a novel and important role of CIP2A expression in autophagy gene expression in HNC.

### HPV16 E6/E7 induce CIP2A overexpression in HNC

**Fig. 6:**
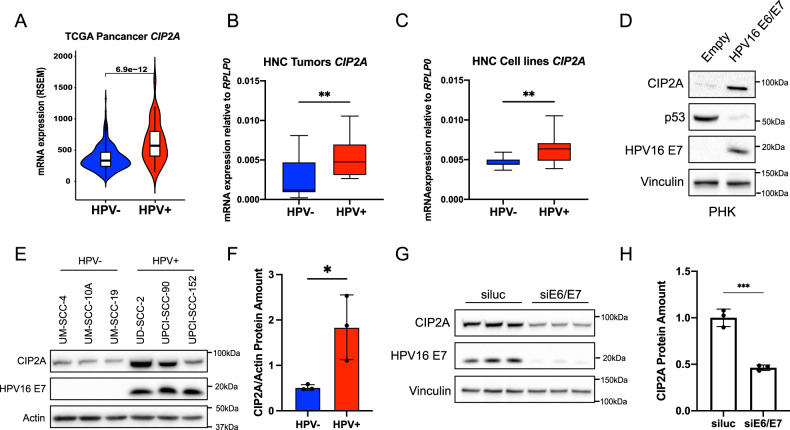
CIP2A is overexpressed in HPV + HNC cells. **A** Violin plot shows a comparison of CIP2A mRNA expression between HPV− (*n* = 415) and HPV+ (*n* = 72) HNC samples using *t*-test. **B** Boxplots show CIP2A mRNA expression relative to RPLP0 between HPV− (*n* = 11) and HPV+ (*n* = 9) HNC tumors from IEO. Whiskers express Tukey. Unpaired *t*-test was performed. **C** Boxplots show CIP2A mRNA expression relative to RPLP0 between HPV− and HPV+ HNC cell lines. Whiskers express Tukey. Unpaired *t*-test was performed (*n* = 6). **D** Western blot shows LC3, c-MYC and CIP2A protein levels in PHK transduced with HPV16 E6/E7 or the empty vector. Vinculin was used as loading control. **E** Western blot shows CIP2A proteins levels in three HPV− (UM-SCC-4, UM-SCC-10A and UM-SCC-19) and three HPV+ (UD-SCC-2, UPCI-SCC-90, UPCI-SCC-152) HNC cell lines. HPV16 E7 was used as a control for HPV+ cell lines. Actin was used as loading control. **F** Histogram shows the densitometric protein levels of CIP2A/Vinculin expressed as mean ± SEM. Unpaired *t*-test was performed. **G** Western blot showing CIP2A and HPV16 E7 protein levels in three biological replicates of UD-SCC-2 cells upon transfection with siE6/E7 or siluc. Vinculin was used as loading control. **H** Histogram shows the normalized densitometric protein levels of CIP2A/Vinculin expressed as mean ±SD. Unpaired *t*-test was performed (*n* = 3).

After demonstrating the importance of CIP2A in c-MYC stability and autophagy in HNC, we evaluated the expression of *CIP2A* in HPV+ vs HPV− HNC cell lines. Upon assessing the mRNA expression of *CIP2A* in the Pancancer dataset, we observed significantly higher expression levels in HPV+ compared to HPV− HNC samples. (Fig. 6A). Interestingly, the same result was confirmed also in primary tumors obtained from IEO hospital (Fig. 6B, S6B), and in HNC cell lines (Fig. 6C).

We then evaluated the possible regulation of HPV16 E6/E7 on CIP2A expression, by transducing PHK with E6/E7 (Fig. 6D). Western blot analysis showed the upregulation of CIP2A in the presence of E6 and E7 proteins, confirmed by higher mRNA levels of *CIP2A* (Supplementary Fig. S6D).

To strengthen our observations, we checked CIP2A protein levels in HPV+ and HPV− HNC cell lines, confirming significant higher levels of CIP2A in the HPV+ subgroup (Fig. 6E, F), and that this upregulation can be significantly reverted upon knock-down of E6/E7 (Fig. 6G, H).

These results are in line with published literature showing overexpression of CIP2A by high-risk HPV due to E7 inhibition of pRb in other systems [36–38]. Along the same line, we also observed the overexpression of *E2F1* (regulated by pRb) both in TCGA cases and in HNC cell lines (Supplementary Fig. S6A, C). Moreover, gene set enrichment analysis (GSEA) performed on RNAseq data from PHK transduced with E6/E7 showed significant enrichment in *E2F1* pathways and E2F targets, as well as *MYC* upregulation, as expected (S6E, S6F, S6G). The same analysis did not show any significant enrichment in mTORC signaling (Supplementary Fig. S6H, I). All these data suggest that HPV indirectly controls *CIP2A* expression activating E2F1 signaling upon pRb inhibition.

### CIP2A and c-MYC downregulation confer resistance to cisplatin in HPV+ HNC cell lines

**Fig. 7:**
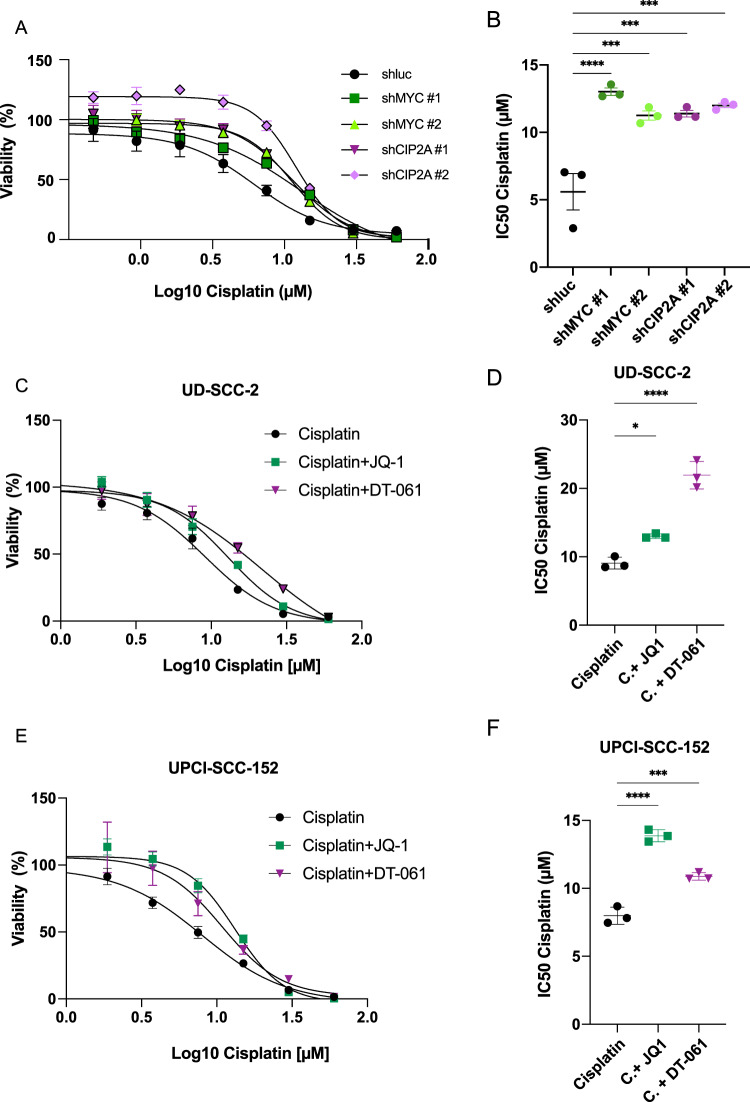
c-MYC downregulation induces cisplatin resistance in HPV+ HNC cells. **A** Dose-response curve of UD-SCC-2 cell lines treated with cisplatin for 72 h upon knock-down of c-MYC, CIP2A or luc. **B** Dot plot showing IC50 of cisplatin in UD-SCC-2 cells upon knock-down of c-MYC, CIP2A or luc. Statistical analysis was performed using one-way ANOVA with Dunnett correction. Bars represent mean ± SD (*n* = 3). **C**, **E** Dose-response curve of UD-SCC-2 (**C**) or UPCI-SCC-152 (**E**) cell lines treated with cisplatin in combination with 1 µM JQ1, 1 µM DT-061 or the vehicle. **D**, **F** Dot plot showing IC50 of cisplatin in UD-SCC-2 (**D**) or UPCI-SCC-152 (**F**) cells treated with 1 µM JQ1, 1 µM DT-061 or the vehicle. Statistical analysis was performed using one-way ANOVA with Dunnett correction. Bars represent mean ± SD (*n* = 3).

Autophagy has a fundamental role in resistance to chemotherapy [39–41]. Recent findings suggest that inhibition of autophagy sensitizes cells to cisplatin treatment [42, 43]. It has been described that HPV+ HNC cells are more sensitive to cisplatin than HPV− cells [44]. To understand the role of c-MYC and CIP2A inhibition in chemosensitivity, we evaluated cisplatin IC50 in the HPV+ UD-SCC-2 cell line upon knock-down of c-MYC or CIP2A. Figure 7A, B shows that the downregulation of either c-MYC or CIP2A induced resistance to cisplatin, with almost a doubling of the IC50 values in UD-SCC-2 cells.

To confirm these results, we downregulated c-MYC pharmacologically, by treating both HPV+ (UD-SCC-2 and UPCI-SCC-152) and HPV− (UM-SCC-4 and UM-SCC-19) cells with the Brd4 inhibitor JQ-1, already known to activate autophagy [45, 46]. We also tested the effects of the PP2A activator DT-061 [47]. JQ-1 induced transcriptional downregulation of *MYC* levels in HPV+ HNC cell lines (Supplementary Fig. S7A, B). To assess the effects of JQ1 and DT-061 on autophagy, we performed multiple assays, including monitoring of LC3-II/LC3-I levels by Western Blot (Supplementary Fig. S7C–E) and LC3-GFP-RFP colocalization (Supplementary Fig. S7F–H). Both treatments were able to induce autophagy in HPV+ HNC cell lines tested and in the UM-SCC-4 HPV− cell line. No significant changes could be detected in the HPV− UM-SCC-19 cells, probably due to low levels of c-MYC (Fig. 3A), suggesting that this cell line only partially relies on c-MYC for its autophagy gene regulation.

We then assessed the IC50 of cisplatin in the presence of 1 µM JQ-1, 1 µM DT-061 or vehicle (DMSO). HPV+ HNC cell lines UD-SCC-2 and UPCI-SCC-152 treated with JQ-1 or DT-061 showed an increased resistance to cisplatin in comparison to the control (Fig. 7C–F). Interestingly, no effects on cisplatin sensitivity could be detected in HPV− HNC cell lines (Supplementary Fig. S7I, J).

Taken together these results suggest the importance of c-MYC activity and autophagy in chemosensitivity, selectively in HPV+ HNC and not in the HPV− HNC subtype.

## Discussion

Autophagy regulation and its role in HPV-related carcinogenesis are far from being well characterized. However, emerging evidence showed the role of HPV16 E6 and E7 oncoproteins as inhibitors of autophagy in various cellular models [30]. Different mechanisms have been proposed, but only a few studies have been conducted in HNC cells [32, 48, 49]. We showed for the first time by electron microscopy that autophagic structures are impaired in HPV+ cell lines compared to HPV− ones. The phenotypic reduction of these structures was due to a wider transcriptional program, in which autophagy and lysosomal biogenesis were inhibited by viral oncoproteins. Specifically, we were able to understand that both HPV16 E6 or E7 oncoprotein alone were sufficient to repress the transcription of autophagy and lysosomal genes.

HPV oncoproteins E6 and E7 have been both described to interact with c-MYC, to upregulate its transcriptional activity and only E6 to also increase its phosphorylated fraction [50, 51]. In this study, we showed a higher activity of c-MYC, confirmed by a higher binding to autophagy gene promoters, that prevented TFEB and TFE3 activity in HPV+ HNC cell lines. The concept of c-MYC preventing TFE3 and TFEB from binding to autophagy and lysosomal genes was previously shown by Annunziata et al., and shed light on how c-MYC can inhibit transcription of autophagy genes [35]. This is also in line with some findings in which the promotion of c-MYC dephosphorylation and degradation upregulates autophagy levels [52].

We showed that c-MYC half-life is significantly different in HPV+ HNC cell lines compared to HPV− ones. However, we could not detect a variation in c-MYC half-life upon knock-down of E6/E7, suggesting that c-MYC stability is not directly regulated by the two oncoproteins. As described above, CIP2A inhibits the effects of PP2A on S62 of c-MYC, important for c-MYC stability and phosphorylation [13]. Here we showed how CIP2A is important in c-MYC stabilization in HPV+ HNC, and its involvement in autophagy inhibition. CIP2A is known to regulate autophagy [53]; however, upon interrogating the TCGA, we assessed for the first time a negative correlation between its expression and autophagy/lysosomal gene expression in HNC patients. Puustinen et al. described that CIP2A inhibits autophagy in a mTORC-dependent manner [54]; however, our results on mTORC involvement are not consistent. Moreover, mTORC inhibition by Torin-1 did not induce a reduction in LC3 colocalized dots, and RNA-seq on PHK did not show any significant enrichment in the mTORC pathway. More studies are needed to better clarify the contribution of this pathway in a CIP2A overexpressed environment.

We demonstrated that the oncoviral proteins E6/E7 upregulated CIP2A mRNA and protein levels in HNC cells and in PHK, in line with Zhang et al. [37]. CIP2A, overexpressed in HPV+ HNC cell lines, in HPV+ cases from the TCGA, and also in primary tumors from HNC patients, could be used as a potential marker for HPV+ tumors. CIP2A upregulation was probably due to E2F1 overactivation, which we also showed in different systems (Supplementary Fig. S6A, C, F, G). E7, mediating pRb impairment, induces the upregulation of E2F1, a known regulator of CIP2A [36–38].

In addition to c-MYC, the other main targets of CIP2A include E2F1, involved in a feedback loop activated by pRb deregulation [38], and Akt. It is worth noting that Akt is not differentially phosphorylated or expressed in HPV+ with respect to HPV− HNC cell lines (Supplementary Fig. S1D), supporting the idea that CIP2A impacts autophagy increasing the activity of c-MYC. However, it would be interesting to conduct further studies aimed at investigating the role of Akt and E2F1 in transcriptional modulation of autophagy in an HPV-related context.

**Fig. 8:**
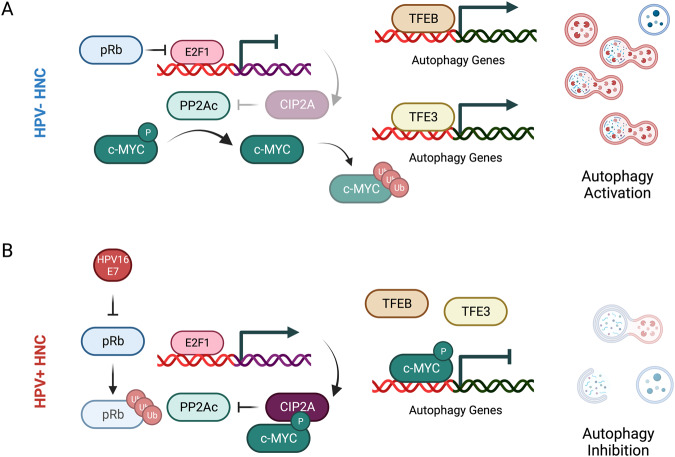
HPV16 E7 induces c-MYC-dependent autophagy inhibition by increasing its stability in HNC. **A**, **B** Proposed working model of c-MYC activity on autophagy in HPV− (**A**) and HPV+ (**B**) HNC cells. Created with Biorender.com.

In HPV− HNC cells E2F activity is tightly regulated, CIP2A is not overexpressed and c-MYC has a short half-life. The expression of autophagy genes is controlled by TFE3 and TFEB and autophagy is functional (Fig. 8A). However, HPV+ HNC cells express high levels of CIP2A due to overactivation of E2F as a consequence of E7-mediated degradation of pRb. This leads to high activity of c-MYC that binds to autophagy and lysosomal gene promoters, resulting in the inhibition of the autophagy pathway (Fig. 8B).

The relevance of these findings was extended to resistance to chemotherapy. As mentioned above, HPV+ HNC cell lines are known to be more sensitive to cisplatin [44]. The notion that autophagy could be important for resistance to chemotherapy [32, 55] was confirmed in this study, in which we showed that downregulation of c-MYC, responsible for autophagy inhibition, resulted in increased resistance to cisplatin. Specifically, both the activation of PP2A using DT-061 and the transcriptional inhibition of c-MYC by JQ-1 successfully modulated autophagy and cisplatin sensitivity. More interestingly, while c-MYC inhibition exerted an effect on cisplatin sensitivity in HPV+ HNC cells, this was not true for HPV− ones, conferring selectivity of the HNC subtype.

The link between HPV, c-MYC and autophagy could give rise to novel approaches to treat HNC patients. Moreover, these results could pave the way to explore how autophagy can be manipulated, to propose more tailored and effective treatments to patients, reducing side effects and toxicity due to high doses of chemotherapy, which are often detrimental for patients.

## Material and methods

### Cell culture

HNC cell lines used in this study [56], were described and cited in previous studies [57, 58], while the laryngeal UM-SCC-17A cell line derives from a laryngeal primary carcinoma of a 48-years old female patient (Millipore). Cells were grown in Dulbecco’s modified Eagle’s medium containing stable glutamine (Euroclone) supplemented with antibiotics, 10% FBS (Fisher Scientific), non-essential amino acids (NEAA) and Plasmocin (Invivogen) to prevent mycoplasma contamination. All experiments, including those monitoring autophagy levels, were performed in the presence of serum. Every 6 months all cell lines were authenticated by short tandem repeat profiling and tested for mycoplasma contamination. Skin biopsies from both male and female donors were collected and anonymized via standardized operative procedures approved by the European Institute of Oncology Ethical Board. Informed consent was obtained from all patients. Adult human epidermal keratinocytes (HKs) were prepared and maintained as previously described [31, 59]. Primary cultures of the isolated cells were then maintained in Keratinocyte Serum-Free Medium (KSFM; Gibco) containing bovine pituitary extract (BPE, 30 μg/mL; Gibco) and epidermal growth factor (EGF, 0.2 ng/mL; Gibco). Cells from passages 2–5 were used for the experiments. All cells were cultured at 37 °C in a 5% CO_2_ buffered incubator.

### Tissue samples

Fresh tumors and adjacent normal tissue samples from both male and female HNC patients were separately collected upon surgery under IEO Biobank approval and preserved at −80 °C. Samples were then snap-frozen in liquid nitrogen and processed for RNA extraction using AllPrep DNA/RNA/protein kit (Qiagen) following manufacturers’ instructions. HPV16 presence was confirmed by qPCR (Supplementary Fig. S6B).

### Transduction, transfection and plasmids

pLXSN HPV16 E6/E7, pLXSN HPV16 E6, pLXSN HPV16 E7 and empty vector were previously described [31]. pLKO.1 PURO shluc and pcDNA3-MYC were a gift from B. Amati (IEO). pcDNA3-CIP2A-v5, pLKO.1 PURO shCIP2A#1, pLKO.1 PURO shCIP2A#1 and pLKO.1 PURO empty vector were a gift from S.Minucci (IEO). pLKO.1 PURO empty vector was subcloned as described in Ravasio et al. [60] using oligos described in the Supplementary Information section to generate pLKO.1 PURO shMYC#1 and pLKO.1 PURO shMYC#2 plasmids. pLKO.1 PURO shCIP2A#1 and pLKO.1 PURO shCIP2A#2 targeting sequences are listed in the Supplementary Information section. pLenti CMV GFP-LC3-RFP was a generous gift from J. Yue (University of Hong Kong, Hong Kong, China). Retroviral and lentiviral transductions were performed as described in Mattoscio et al. [31]. HNC cell lines were transfected with Lipofectamine 3000 (Thermo Fisher Scientific) to overexpress pcDNA3 MYC, pcDNA3 CIP2A-v5 plasmids, or the empty vector. siRNAs listed in the Supplementary Information section were transfected using RNAiMAX reagent (Thermo Fisher Scientific), following manufacturers’ instructions. After 6 h from transfection cells were detached and seeded on glass coverslips and cell culture dishes and harvested for further analyses after 72 h from transfection (IF, western blots, qPCR). qPCR was used to confirm HPV16 E6 and E7 downregulation (Fig. 2E).

### Drugs and treatments

Torin-1 was purchased by Selleckchem at concentrations specified in the figure legends. Bafilomycin A1 was purchased by Sigma-Alldrich and used at 10 nM concentration. Cycloheximide was purchased from Sigma-Aldrich and used at 25 µg/mL concentration for the time specified in figure legends. DT-061 and JQ-1 were purchased by Medchem Express and used as specified in the figure legends.

### Electron microscopy

HNC cells were plated in a growth medium at a density of 10^5^ cells/well in 6-well plates and grown for 24 h. Cells were fixed in 2.5% glutaraldehyde (Sigma-Aldrich) solution for 1 h at room temperature. After fixation cells were rinsed with 0.1 M sodium cacodylate (Sigma-Aldrich) buffer (pH 7.4), three times for 5 min at room temperature. Cells were post-fixed in a solution containing 1% OsO_4_ (Sigma-Aldrich) 1.5% K_4_Fe(CN)_6_ (Sigma-Aldrich), and 0.1 M sodium cacodylate for 1 h at 4 °C protected from light. After fixation samples were washed 3 times for 5 min with 0.1 M sodium cacodylate buffer and washed 5 times with distilled water. Samples were then stained with 0.5% uranyl acetate at 4 °C overnight, protected from light. After 5 washing steps with distilled water, samples were dehydrated using the following increasing concentration of ethanol (5 min per step): 30%, 50%, 70%, 80%, 90 and 96%. Then, samples were incubated 3 times for 5 min in 100% ethanol. After dehydration, samples were covered with a mixture of ethanol and epoxy resin (1:1) and left for 2 h on a shaker at room temperature. After 2 changes of pure epoxy resin (1 h per change) at room temperature, samples were finally embedded in fresh epoxy resin and polymerized overnight at 45 °C and then incubated at 60 °C for 24 h.

Ultrathin sectioning (70 nm) was performed on a Leica EM UC6 ultramicrotome. Sections were picked up and positioned on a 300 mesh squares grid. Thin sections were contrasted with 2% aqueous uranyl acetate for 5 min, followed by 3 washes in filtered distilled water (1 min each). Subsequently, grids were placed in a drop of Sato lead stain and incubated for 2 min, rinsed 3 times with pure water and dried with Whatman filter paper. Images were collected using a FEI Tecnai-12 transmission electron microscope.

Autophagic structures, termed autophagic vacuoles (AVs), which are collectively referred to as autophagosomes, amphisomes, and autolysosomes were quantified using Image J software. Multivesicular bodies, endosomes, and phagophores were not included in the analysis. Quantification was performed in terms of the number of AVs per cellular profile and in the area occupied by AVs.

### Confocal microscopy

Cells expressing LC3-GFP-RFP were seeded on coverslips and incubated for 24 h at 37 °C. cells were treated with 1 µM Torin-1 (Selleckchem), 10 nM Bafilomycin A1 (Sigma-Aldrich), 10 µM JQ-1 (Medchem Express), 10 µM DT-061 (Medchem Express) or vehicle for 24 h, fixed with 4% PFA (Sigma-Aldrich). Nuclei were stained with DAPI. Coverslips were then mounted, and images were taken using a Leica SP8 AOBS (Leica microsystems) confocal microscopy using 63X oil objective and acquired with Leica Confocal Software. Quantification of dots number and colocalization were measured adapting the previously reported Red and Green Puncta Colocalization plugin and macro of ImageJ software [61].

Cells transfected with plasmids or siRNA were plated on glass coverslips were fixed with ice-cold methanol/acetone (1:1) for 2 min at −20 °C, washed and blocked with 5% BSA for 30 min. Cells were then incubated with primary antibodies for 45 min at room temperature. The following antibodies (specified in the Supplementary Information section) were used: anti-LC3 1:200, anti-LAMP2 1:100. Next, cells were incubated with Alexa Fluor (Life technologies) 488 or 647 fluorescent dye-conjugated secondary antibodies for 30 min at room temperature. Nuclei were counterstained with DAPI, mounted and analyzed by confocal microscopy Leica SP8 AOBS (Leica microsystems).

### Cell lysis and Western blotting

Cells were lysed in a sodium dodecyl sulfate (SDS) lysis previously described [58]. After lysis, an equal amount of protein for each sample was resuspended in denaturing sample loading buffer, separated on SDS-PAGE gel and immunoblotted with the indicated antibodies. The list of antibodies is reported in the Supplementary Information section. Membranes were then incubated with the appropriate horseradish peroxidase (HRP) secondary antibodies and the signal was acquired with Chemidoc (Bio-Rad). Densitometric analysis of the intensity of the protein bands was performed using ImageLab software (Bio-Rad).

### RT-qPCR

RNA was extracted from cells with the Quick-RNA MiniPrep kit (Zymo research). cDNA was generated by reverse transcription with Lunascript RT Supermix (New England Biolabs). Relative levels of specific mRNAs were determined with the Luna Universal qPCR master mix (New England Biolabs). All PCR reactions were performed with a QuantStudio 6 Fast Real-Time PCR system (Thermo Fisher Scientific). Neutral Ribosomal Phosphoprotein P0 (RPLP0) was used as a house-keeper gene for normalization. mRNA expression was calculated using the 2^−^^∆CT^ or 2^−^^∆∆CT^ methods. Primers used are listed in the Supplementary Information section.

### Chromatin immunoprecipitation (ChIP)

In this, 10^7^ UD-SCC-2 and UPCI-SCC-152 cells treated with 250 or 500 nM Torin-1 for 16 h were fixed with 1% formaldehyde for 10 min at RT. Then, 125 µM glycine was used to quench formaldehyde. After 3 PBS washes cells were harvested, centrifuged at 300 rcf and lysed using Lysis Buffer 1 (50 mM Hepes, pH 7.5, 140 mM NaCl, 1 mM EDTA, 10% glycerol, 0.5% NP-40, 0.25% Triton X-100) in the presence of protease inhibitors. After 10 min on ice cells were centrifuged at 300 rcf and resuspended in Lysis Buffer 2 (10 mM Tris-Hcl pH8, 200 mM NaCl, 1 mM EDTA, 0.5 mM EGTA) in the presence of protease inhibitors and rocked gently for 10 min at RT. After centrifugation (300 rcf) nuclei were lysed in Lysis Buffer 3 (10 mM Tris-Hcl pH8, 100 mM NaCl, 1 mM EDTA, 0.5 mM EGTA, 0.1% Sodium Deoxycholate, 0.5% N-laureylsarcosine). Chromatin was sheared by sonication, to obtain a bulk size of 200–500 bp. Chromatin proteins were then quantified using DC assay (Bio-Rad). Then, 500 or 1000 µg chromatin was incubated overnight at 4 °C with Protein G Dynabeads (Invitrogen) coupled with 10 μg of antibody. After immunoprecipitation, beads were recovered using a magnet and washed; chromatin was eluted using TE containing 2% SDS and cross‐links reverted overnight at 65 °C. DNA was purified with QIAquick columns (Qiagen). ChIPs were performed in triplicate or duplicates for antibodies and in duplicate for IgGs. Antibodies used are listed in the Supplementary Information section. Next, 2 µL of the immunoprecipitated material or the purified input was used in qPCR reactions using the Luna Universal qPCR master mix (New England Biolabs) with primers listed in the Supplementary Information section. All PCR reactions were performed with a QuantStudio 6 Fast Real-Time PCR system (Thermo Fisher Scientific). The values shown in the graphs are expressed as fold enrichment of the antibody to the IgGs.

### Cell viability assay

UD-SCC-2, UM-SCC-4, UM-SCC-19 and UPCI-SCC-152 cell lines were seeded into 96-well white plates (1500 or 2000 cells/well) and 24 h later were treated with 9 different concentrations of cisplatin (Teva) for 72 h and vehicle, alone or in combination with 1 µM JQ-1 (Medchem Express) or 1 µM DT-061 (Medchem Express). UD-SCC-2 cell lines transduced with shluc, shMYC#1, shMYC#2, shCIP2A#1, shCIP2A#2 were seeded into 96-well white plates (2000 cells/well) and 24 h later were treated with 9 different concentrations of cisplatin (Teva) for 72 h. Cell viability was then assessed using CellTiter-Glo Luminescent Cell Viability Assay (Promega) following manufacturer instructions and luminescence was measured using a Glomax Discover plates reader (Promega).

### RNA‐seq data analysis

RNA form PHK transduced with E6, E7, E6/E7 or the empty vector was extracted from cells with the Quick-RNA MiniPrep kit according to the manufacturer’s instructions (Zymo Research). RNA quality and integrity were assessed with an Agilent 2100 bioanalyzer using an RNA 6000 Nano Kit (Agilent Technologies). mRNA-Seq library was prepared from 500 ng of total RNA with the TruSeq Stranded Total RNA (Illumina) and sequenced with Novaseq 6000 (Illumina) with a read length of 50 bp (paired-end). FastX-toolkits and FastQC tools were used to filter reads and control the quality of all raw data; TopHat was used for the alignment of reads to the reference genome. Alignment was performed using HTS-flow [62]. A paired differential expression analysis was performed using the Bioconductor DESeq2 R package [63]. Genes were identified as DEGs when the false discovery rate (FDR) ≤0.05. Gene Ontology (GO) enrichment analyses were performed using the SRplot tool [64, 65]. Gene set enrichment analysis (GSEA) [66] was performed using all expressed genes ranked by signed *P*‐value and 1000 gene set permutations.

### Statistical analysis of TCGA data

RSEM normalized expression data was extracted from the TCGA PanCancer Atlas on the cBioPortal [67, 68]. Patient samples with known HPV status were grouped as HPV+ and HPV−. This resulted in 72 HPV+ and 415 HPV− samples with data available for the HNC gene expression analysis. Violin plot comparisons of gene expression and statistical analysis were performed by https://www.bioinformatics.com.cn/en, a free online platform for data analysis and visualization.

### Statistical analysis

Statistical analysis specified in figure legends was evaluated using GraphPad Prism v9.0 (Graphpad Software, Inc., San Diego, California, USA). *P*-value is represented as *p < 0.05, **p < 0.01, ***p < 0.001, ****p < 0.0001, n.s. not significant.

### Graphs and figures

Graphs were plotted using either GraphPad Prism v9.0 (Graphpad Software, Inc., San Diego, California, USA) or https://www.bioinformatics.com.cn/en, a free online platform for data analysis and visualization. Figures and panels were designed using Affinity Designer. Some figures were created with Biorender.com.

### Supplementary information

Supplementary Figure Legends

Figure S1

Figure S2

Figure S3

Figure S4

Figure S5

Figure S6

Figure S7

Supplementary Information

Reproducibility Checklist

Original Blots

## Data Availability

All NGS data of this study (raw and processed) were deposited in the gene expression omnibus (GEO) database under the accession number GSE235662. All relevant data are within the paper and its Supporting Information files, further inquiries can be directed to the corresponding author. Original uncropped western blots can be found in the “Original Blots” supplemental file.
