# Supplementary material for: c-MYC-dependent transcriptional inhibition of autophagy is implicated in cisplatin sensitivity in HPV-positive head and neck cancer
Source: Cell Death Dis. 2023 Nov 4;14(11):719. doi: 10.1038/s41419-023-06248-3 (PMC10625625; doi:10.1038/s41419-023-06248-3)
Supplement: Supplementary file 1 — Supplementary Figure Legends [file 41419_2023_6248_MOESM1_ESM.docx]

**Supplementary Figures**

**Figure Legends**

**Supplementary Figure S1 – Autophagy levels in HNC cell lines.** A) Quantification of EM. Box plots show the area occupied by AVs per cellular profile (n>19). Whiskers represent Tukey analysis. Statistical analysis was performed using ordinary one-way ANOVA corrected comparing False Discovery Rate (two-stage step-up method of Benjamini, Krieger and Yakuteli). B, C, D, E) Western blot shows Beclin1 (B), p62 (C), p-Akt/Akt (D) or p-p70S6K/p70S6K (E) protein levels in three HPV- (UM-SCC-4, UM-SCC-10A and UM-SCC-19) and three HPV+ (UD-SCC-2, UPCI-SCC-90, UPCI-SCC-152) HNC cell lines. HPV16 E7 is HPV+ control. P-Akt/Akt ratio is reported (D). Vinculin was used as housekeeping protein.

**Supplementary Figure S2 – Effects of HPV16 E6 and E7 on autophagy in PHK.** A) Western blot shows LC3, p53 and HPV16 E7 protein levels in PHK transduced with HPV16 E7, E6/E7 or the empty vector. Vinculin was used as loading control. LC3 protein amount is shown as LC3-II/LC3-I. B) Relative mRNA levels of HPV16 E6 and E7 genes in PHK transduced with HPV16 E7, E6/E7 or the empty vector. C) Boxplots showing mRNA expression of autophagy and lysosomal genes in PHK transduced with E7, E6/E7, or the empty vector. Whiskers represent Tukey. Statistical analysis was performed using ordinary one-way ANOVA with Dunnett’s correction (n=8). D) Heatmap showing Differentially expressed genes from RNA-seq analysis in PHK transduced with HPV16 E6, HPV16 E7 or HPV16 E6/E7 with respect to empty vector (n=4). E) Heatmap of autophagy and lysosomal gene differentially expressed in PHK from panel D. F) GO (Gene Ontology) Cell compartment and KEGG pathway enrichment bubble plot of genes downregulated by HPV16 E6 from panel D. G) GO (Gene Ontology) Cell compartment and KEGG pathway enrichment bubble plot of genes downregulated by HPV16 E7 from panel D.

**Supplementary Figure S3 – TFEB, TFE3 and c-MYC expression in HNC cell lines.** A, B, C) Histograms show RT-qPCR results of UM-SCC-4, UM-SCC-19, UD-SCC-2 and UPCI-SCC-152 cell lines, representing mRNA expression of TFEB (A), TFE3 (B), and MYC (C) genes relative to the housekeeping gene (RPLP0) using 2^(-dCT) method. Statistical analysis was performed using ordinary one-way ANOVA with Bonferroni correction. Bars represent means ±SD (n=3). D) Histograms represent TFEB (n=3), TFE3 (n=3), and c-MYC (n=3) binding to the promoters of *GLA,* and *SQSTM1, and ATP6V1H* expressed as fold enrichment to the IgG in UD-SCC-2 or UM-SCC-4 cell lines. Statistical analysis was performed using two-way ANOVA corrected comparing False Discovery Rate (two-stage step-up method of Benjamini, Krieger and Yakuteli). Bars express means ±SD. E-G) Histogram represents TFEB binding to the promoter of *GLA* (E)*,* *SQSTM1* (F), and *ATP6V1H* (G), expressed as fold enrichment to the IgG in UD-SCC-2 transduced with shMYC or shluc. Statistical analysis was performed using t test (n=2). Bars express means ±SD.

**Supplementary Figure S4 – Impact of c-MYC on autophagy in HNC cell lines.** A, B) Western blot showing LC3 regulation upon knock-down of c-MYC in UD-SCC-2 (A) or UPCI-SCC-90 (B) cell lines. LC3-II/LC3-I ratio is reported. Vinculin is used as loading control. C) Western blot showing LC3 regulation upon overexpression of c-MYC in UPCI-SCC-152 cells. LC3-II/LC3-I ratio is reported. Vinculin is used as loading control. D) Boxplots showing mRNA expression of autophagy genes in UD-SCC-2 cells transduced with shMYC compared to the shluc. Whiskers express Tukey analysis. Statistical analysis was performed using t test (n=7). E) Western blots show c-MYC protein levels at different time points (expressed in minutes) after cycloheximide treatment (CHX) in HPV- HNC cell lines (UM-SCC-4 and UM-SCC-17A). Vinculin or GAPDH are used as housekeeping. F) Western blots show c-MYC protein levels at different time points (expressed in minutes) after cycloheximide treatment (CHX) in HPV+ HNC cell lines (UM-SCC-104 and UPCI-SCC-152). GAPDH is used as housekeeping.

**Supplementary Figure S5 – CIP2A overexpression and autophagy.** A,B,C) Extended Scatter plots express the Pearson correlation (red line) between mRNA expression of CIP2A and autophagy/lysosomal genes (RSEM) in HNC samples within the Pancancer dataset from the TCGA.

**Supplementary Figure S6 – E2F1 overexpression activation and expression in HPV+ cases.** A) Violin plot shows comparison of E2F1 mRNA expression between HPV- (n=415) and HPV+ (n=72) HNC samples within the Pancancer dataset from the TCGA. Statistical analysis was performed using unpaired t test. B) Box plots show HPV16 E7 mRNA expression relative to RPLP0 between HPV- (n=11) and HPV+ (n=9) HNC tumors from IEO. Whiskers express Tukey. Unpaired t test was performed. C) Box plots show E2F1 mRNA expression relative to RPLP0 between HPV- and HPV+ HNC cell lines. Whiskers express Tukey. Unpaired t test was performed (n=6). D) Box plots show CIP2A relative mRNA expression in PHK transduced with HPV16 E6/E7 or the empty vector. Whiskers express Tukey. Unpaired t test was performed (n=6). E,F, G, H, I) GSEA plot of gene set MYC_UP (E), E2F1_UP (F), MTOR_UP (I) from KEGG Pathway analysis, E2F_TARGETS (G), MTORC1_SIGNALLING (H) from Hallmark cancer analysis performed on RNA-seq data from PHK transduced with E6/E7 compared to the empty vector. Normalized Enrichment Score (NES), p-value and FDR are expressed.

**Supplementary Figure S7 – Effects of combination of Cisplatin with JQ1 or DT-061 in HNC cell lines.** A,B) Box-plots showing mRNA expression of MAP1LC3B or MYC in UD-SCC-2 (A), and UPCI-SCC-152 (B) cells treated with 10µM JQ1 for 24h. Whiskers express Tukey. Statistical analysis was performed using t test (n=4). C) Western blot shows LC3 levels in two HPV- (UM-SCC-4, UM-SCC-19), and two HPV+ (UD-SCC-2, UPCI-SCC-152) HNC cell lines upon treatment with JQ-1 10µM for 24h. LC3-II/LC3-I ratio is reported. Vinculin was used as loading control. D) Western blot shows LC3 levels in UM-SCC-4 and UM-SCC-19 HNC cell lines upon treatment with DT-061 10µM for 6h. LC3-II/LC3-I ratio is reported. Vinculin was used as loading control. E) Western blot shows LC3 levels in UD-SCC-2 and UPCI-SCC-152 HNC cell lines upon treatment with DT-061 10µM for 6h. LC3-II/LC3-I ratio is reported. Vinculin was used as loading control. F) Confocal micrographs of HPV- (UM-SCC-4) and HPV+ (UPCI-SCC-152) cell lines transduced with GFP-RFP-LC3 (Red and Green dots) and treated with 1µM DT-061, 1µM JQ-1 or vehicle (DMSO) for 24h. Nuclei were stained with DAPI (Blue). G,H) Quantification of colocalized dots for each RFP dot analysis of (G) HPV- (UM-SCC-4, UM-SCC-19) and (H) HPV+ (UD-SCC-2, UPCI-SCC-152) cell lines transduced with GFP-RFP-LC3 (Red and Green dots) and treated with 1µM DT-061, 1µM JQ-1 or vehicle (DMSO) for 24h. Each dot represents a different field. Statistical analysis was performed using one-way ANOVA test with Dunnett’s correction (n=10). Bars are expressed as mean ±SD. I) Dose-response curve of UM-SCC-4 and UM-SCC-19 cell lines treated with Cisplatin in combination with 1µM JQ1, or the vehicle (n=3). J) Dose-response curve of UM-SCC-4 and UM-SCC-19 cell lines treated with Cisplatin in combination with 1µM DT-061, or the vehicle. Bars represent mean ±SD (n=3).
