# Supplementary figures and images for: c-MYC-dependent transcriptional inhibition of autophagy is implicated in cisplatin sensitivity in HPV-positive head and neck cancer
Source: Cell Death Dis. 2023 Nov 4;14(11):719. doi: 10.1038/s41419-023-06248-3 (PMC10625625; doi:10.1038/s41419-023-06248-3)

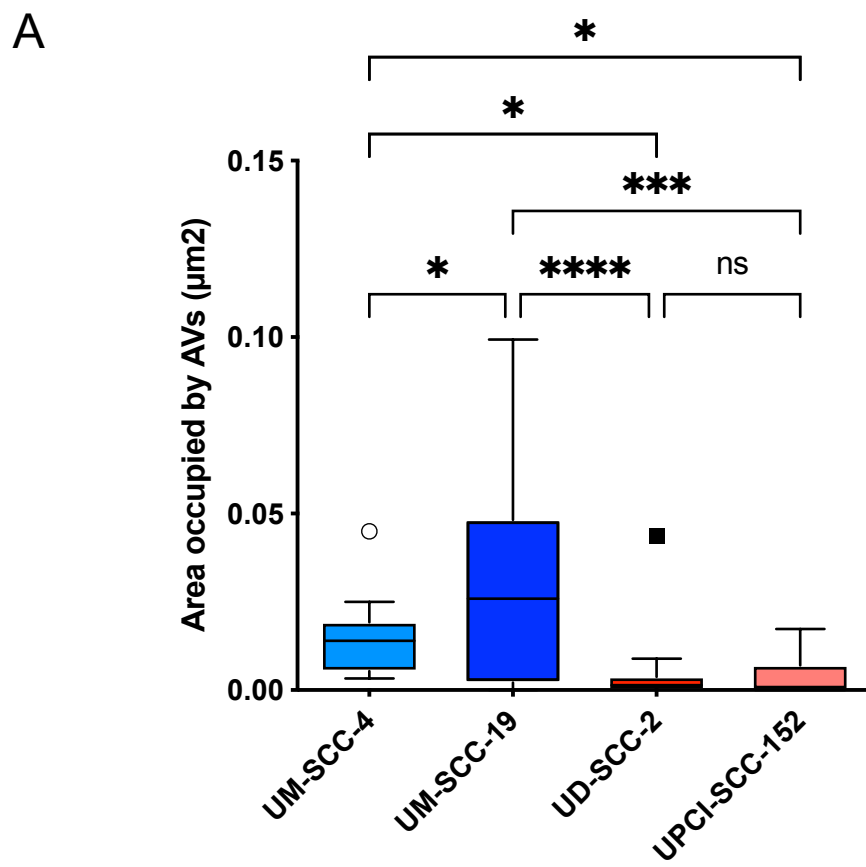

**B**

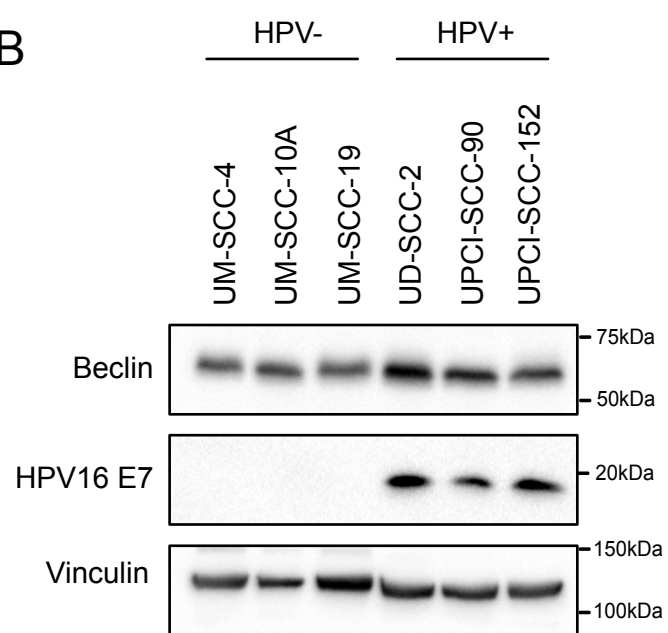

**C**

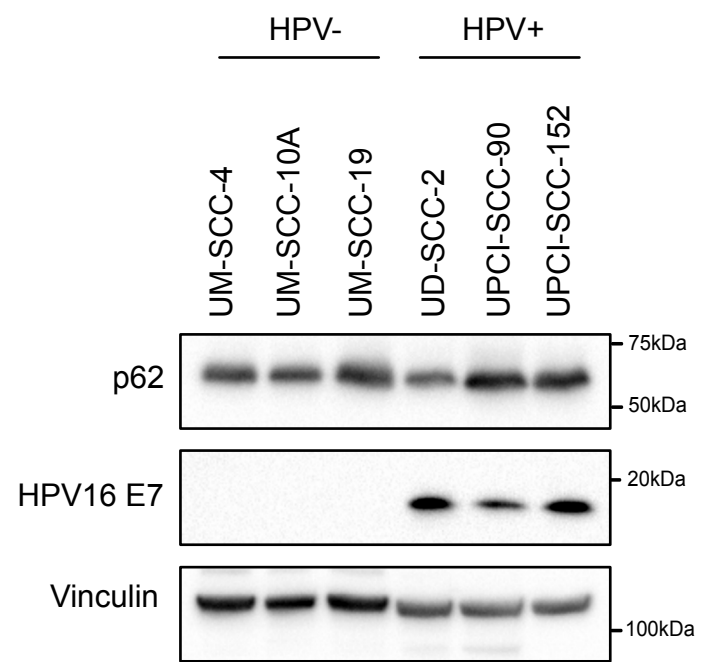

**D**

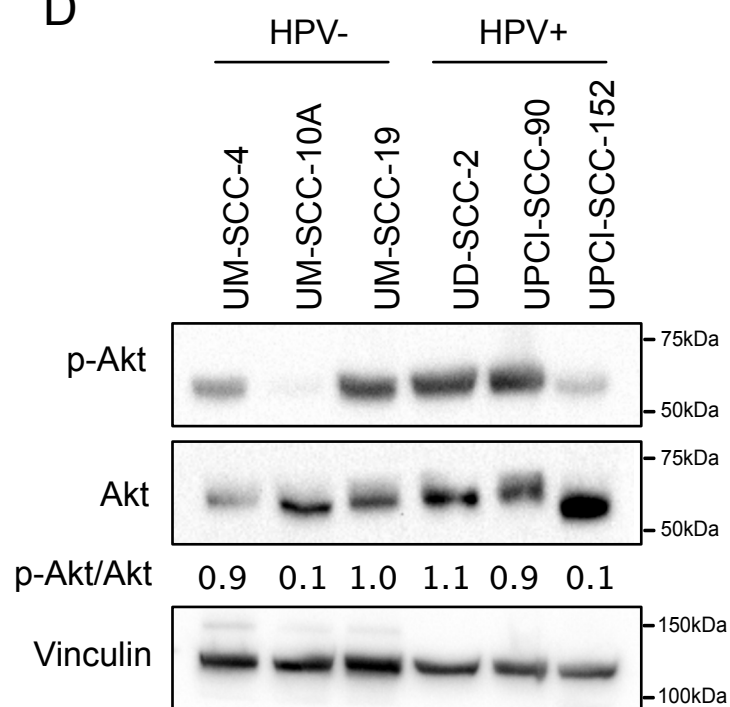

**E**

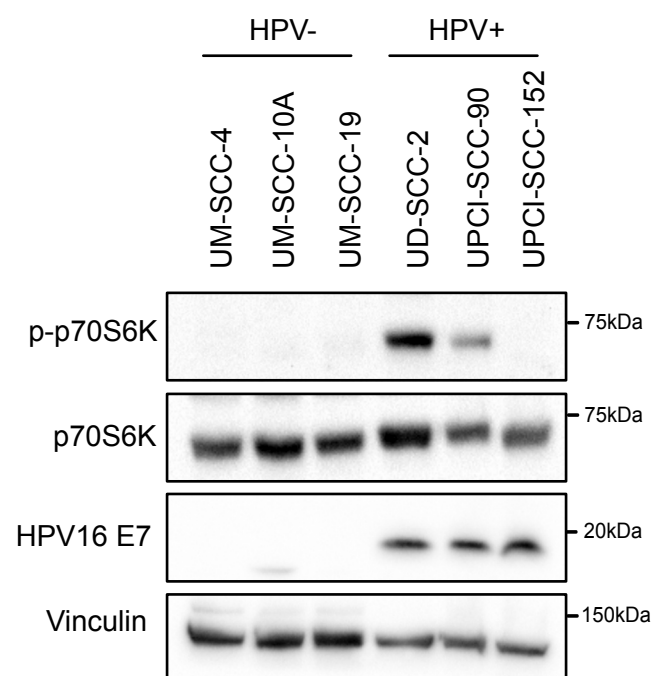

Supplement: Supplementary file 2 — Figure S1 [file 41419_2023_6248_MOESM2_ESM.pdf]

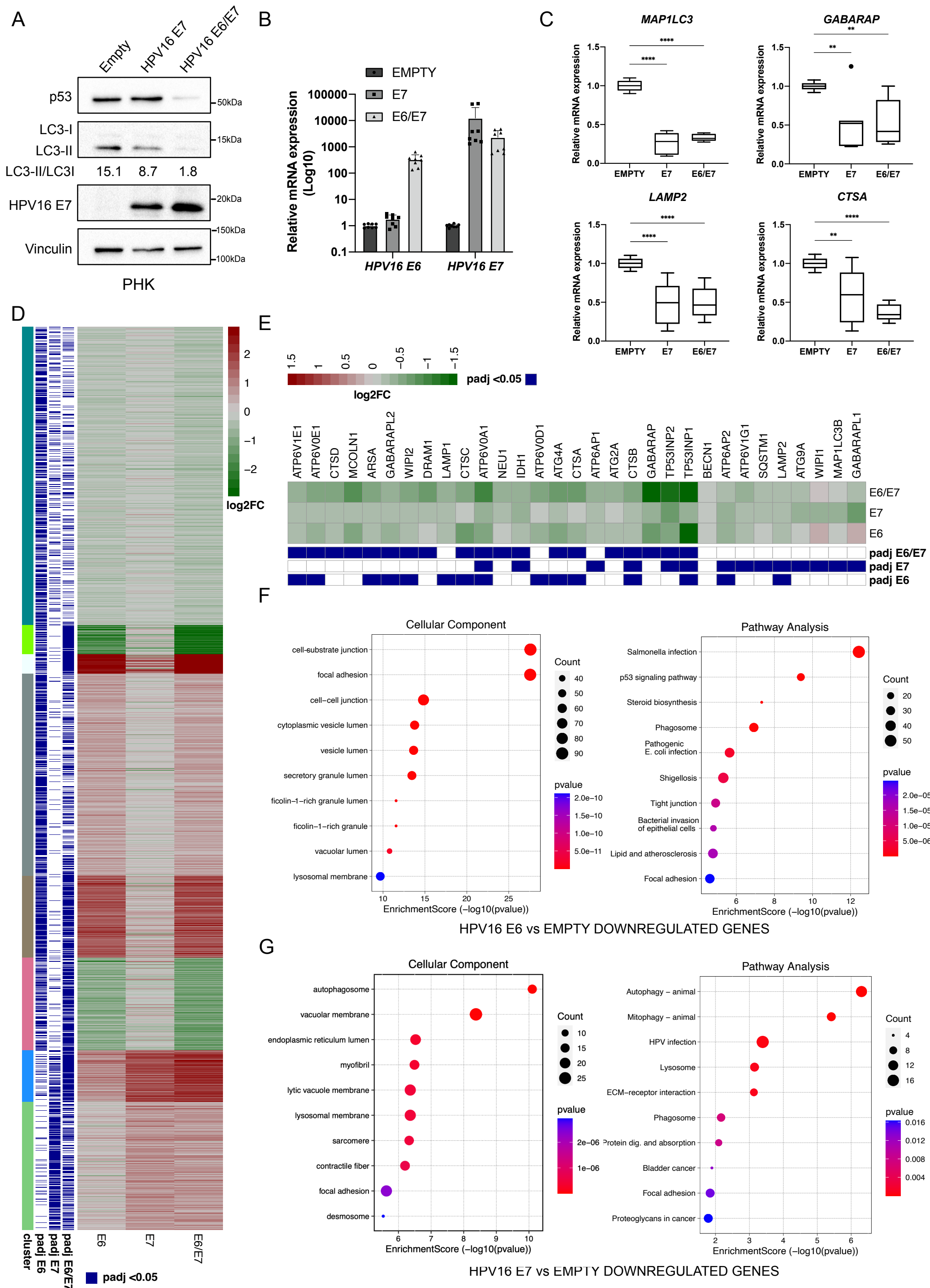

Supplement: Supplementary file 3 — Figure S2 [file 41419_2023_6248_MOESM3_ESM.pdf]

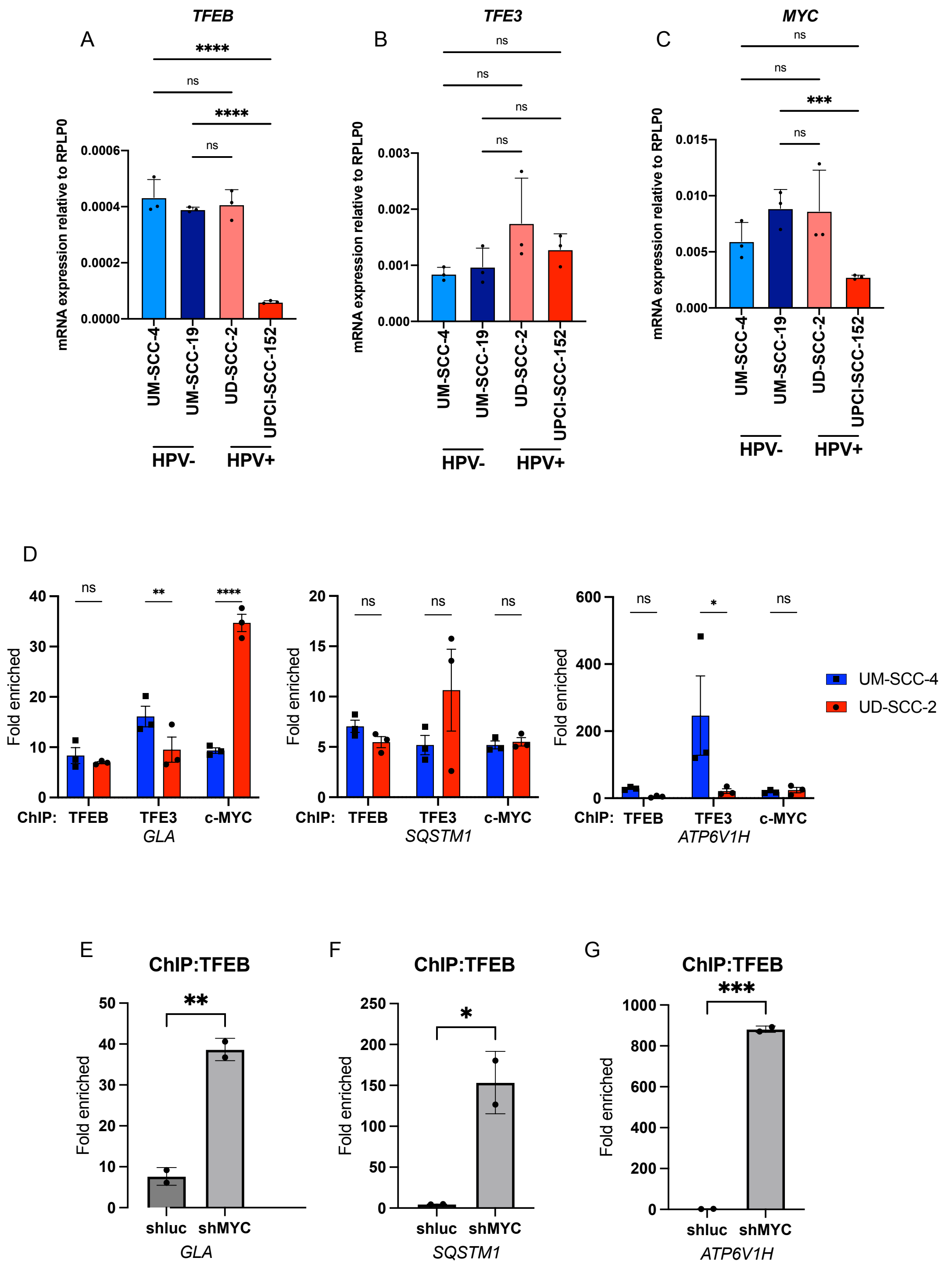

Supplement: Supplementary file 4 — Figure S3 [file 41419_2023_6248_MOESM4_ESM.pdf]

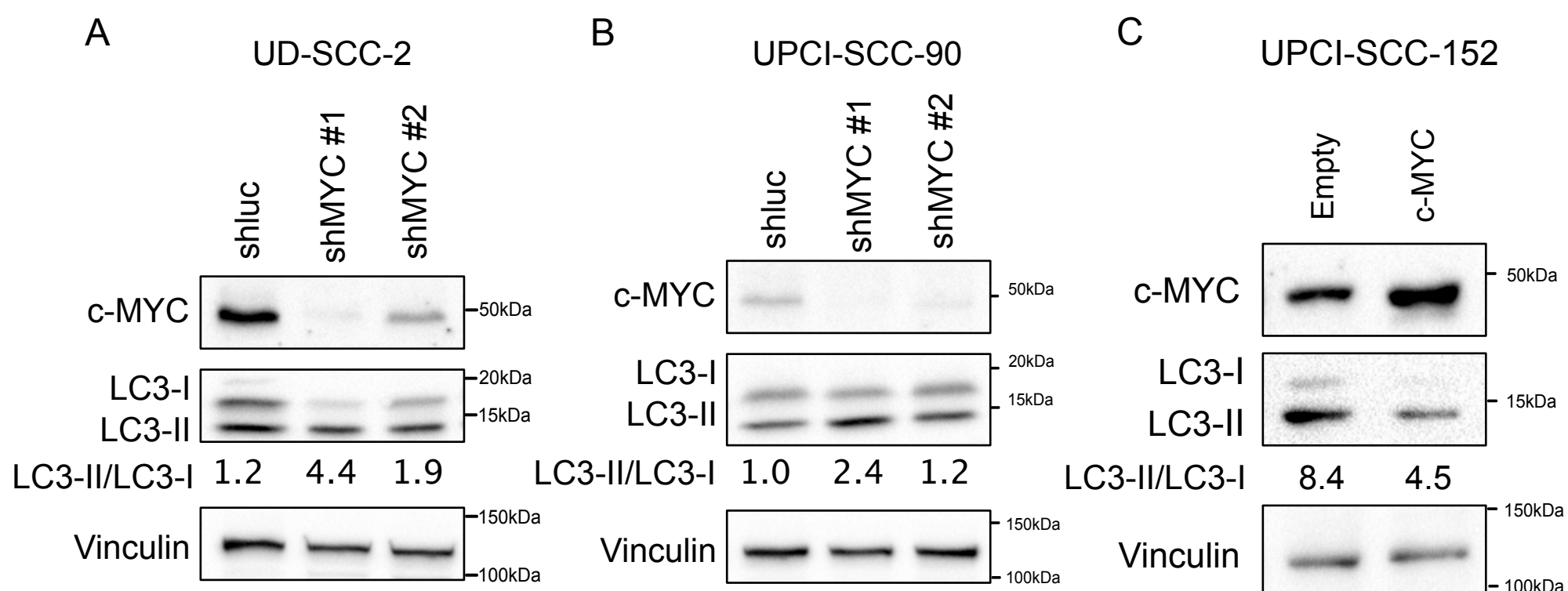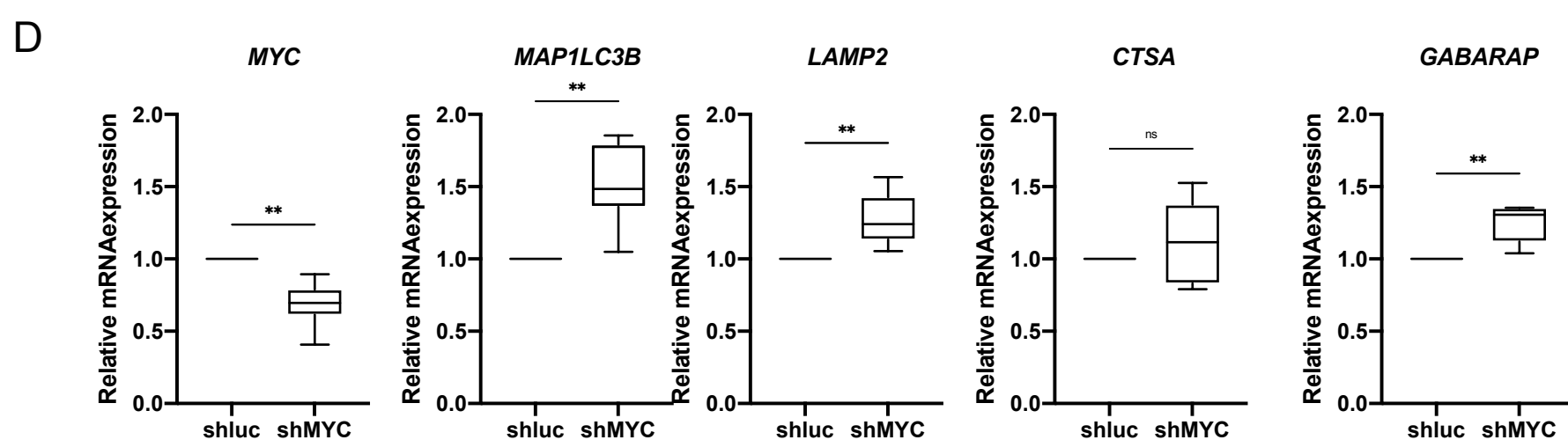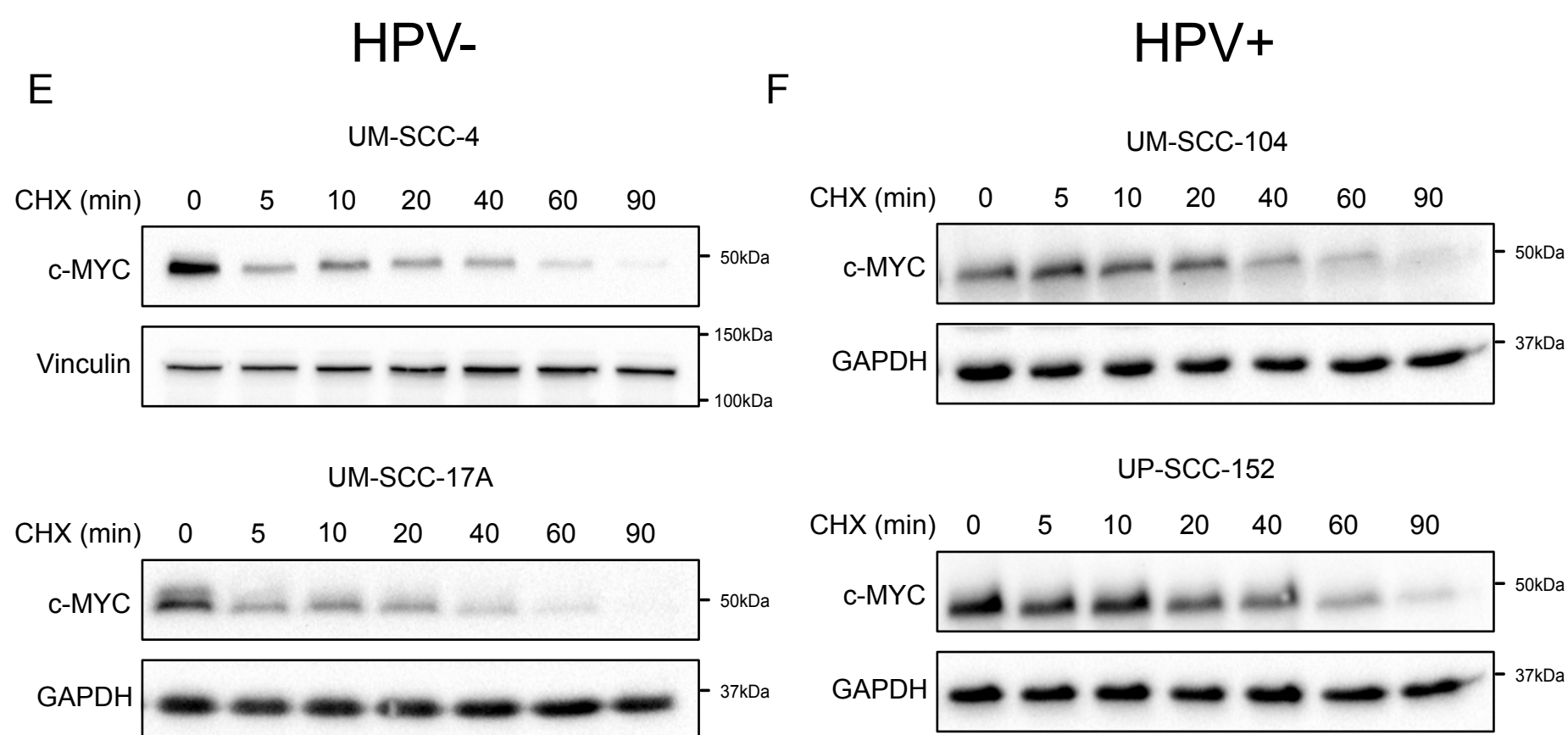

Supplement: Supplementary file 5 — Figure S4 [file 41419_2023_6248_MOESM5_ESM.pdf]

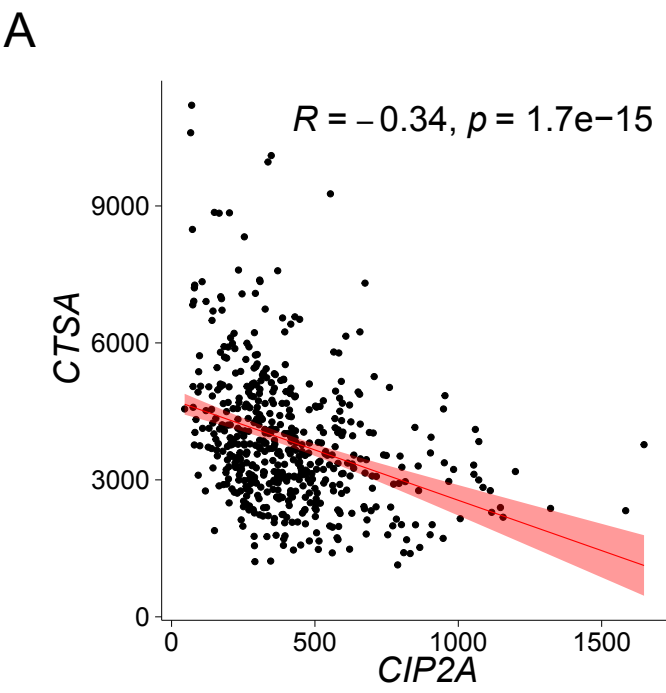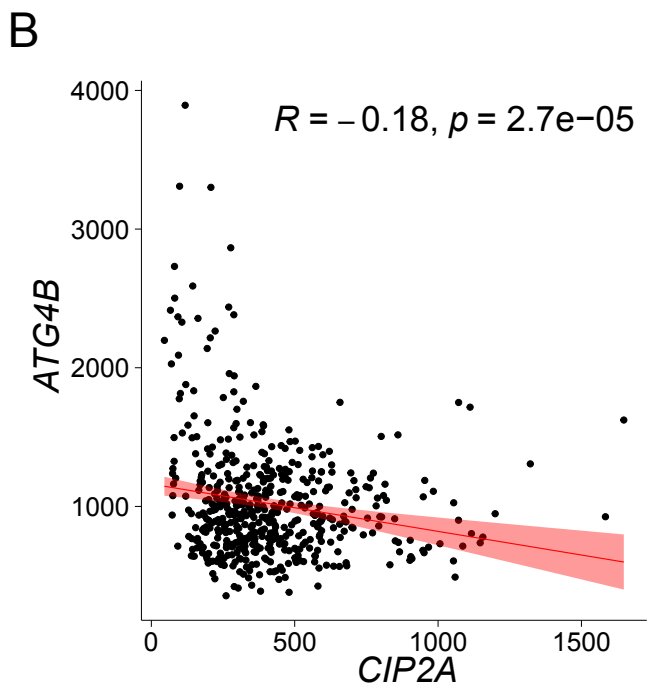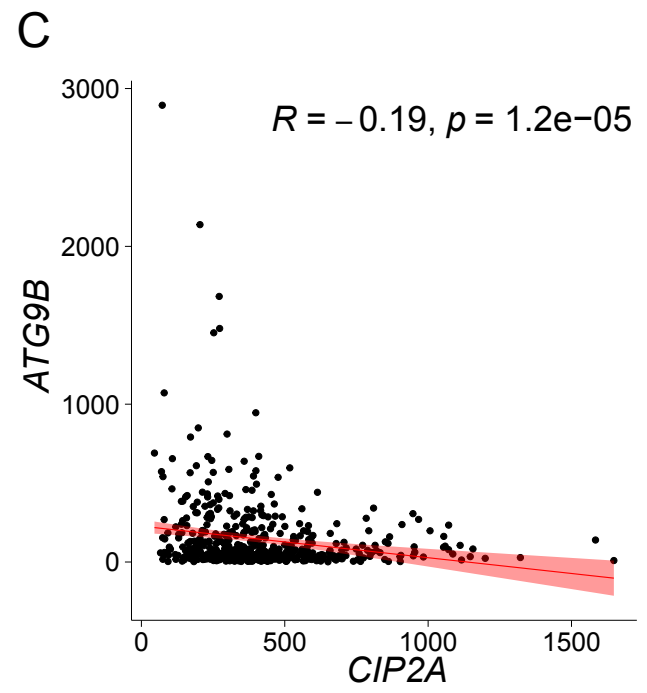

Supplement: Supplementary file 6 — Figure S5 [file 41419_2023_6248_MOESM6_ESM.pdf]

Figure S6

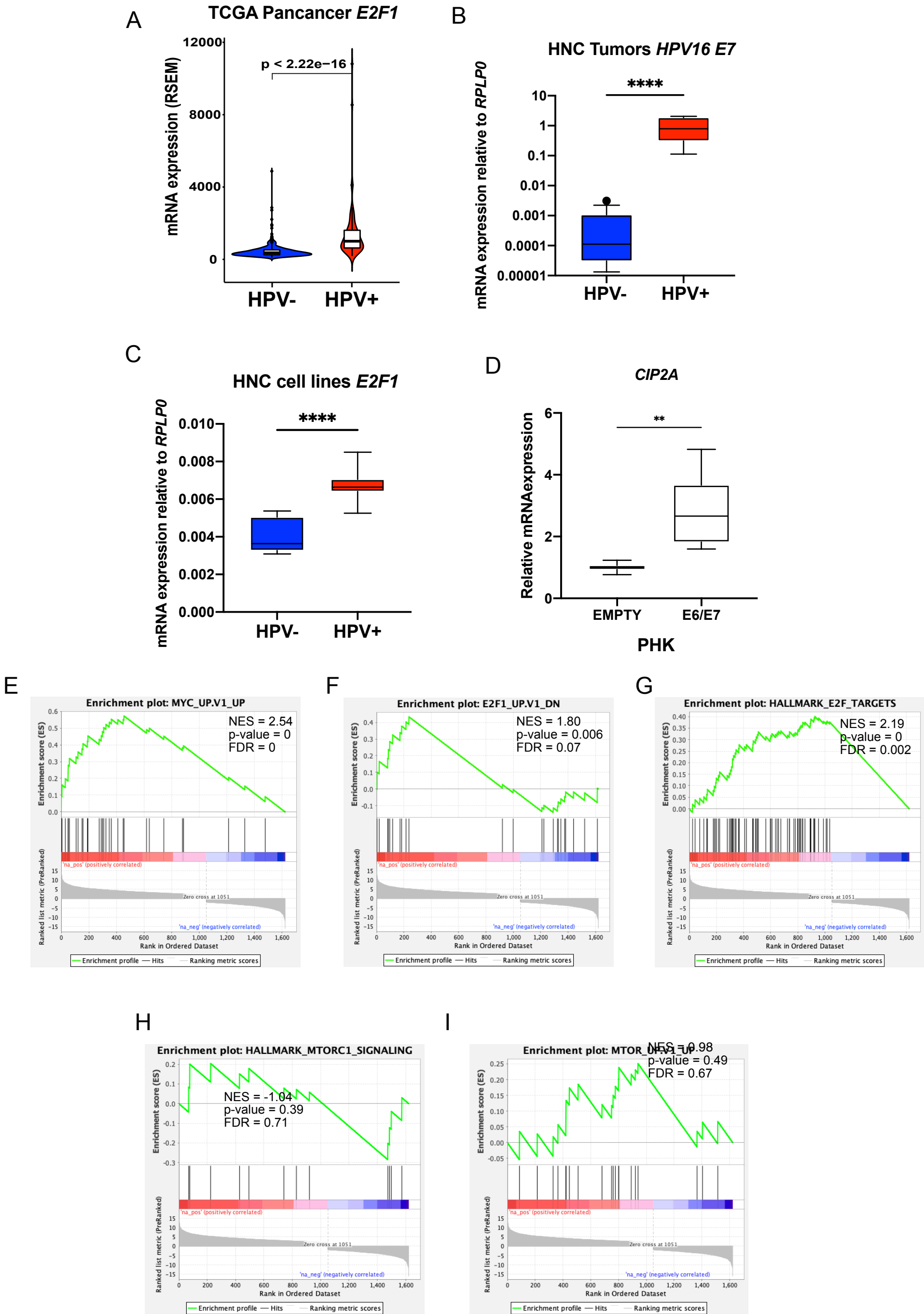

Supplement: Supplementary file 7 — Figure S6 [file 41419_2023_6248_MOESM7_ESM.pdf]

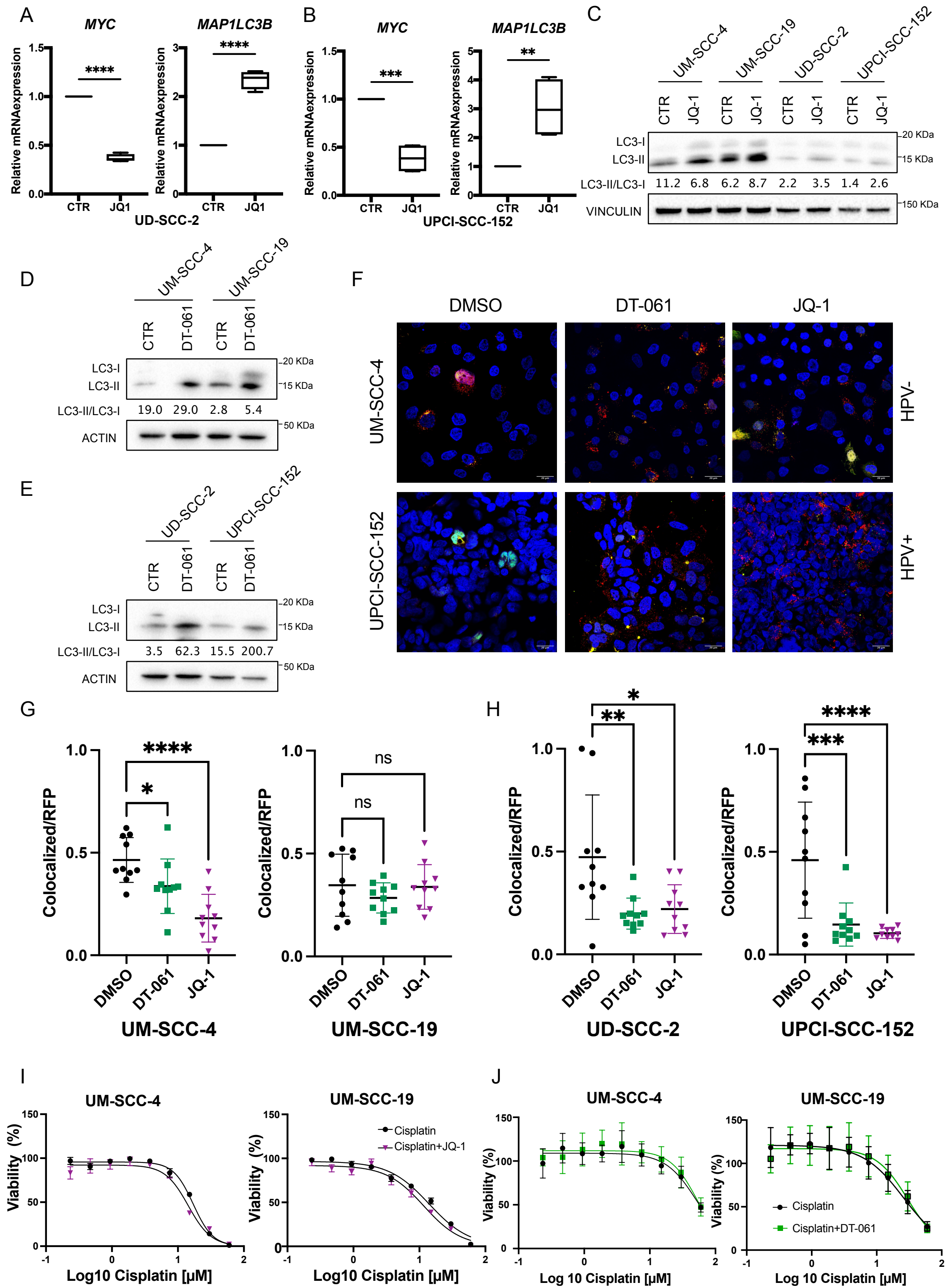

Supplement: Supplementary file 8 — Figure S7 [file 41419_2023_6248_MOESM8_ESM.pdf]
