# Supplementary material for: c-MYC-dependent transcriptional inhibition of autophagy is implicated in cisplatin sensitivity in HPV-positive head and neck cancer
Source: Cell Death Dis. 2023 Nov 4;14(11):719. doi: 10.1038/s41419-023-06248-3 (PMC10625625; doi:10.1038/s41419-023-06248-3)
Supplement: Supplementary file 9 — Supplementary Information [file 41419_2023_6248_MOESM9_ESM.docx]

**Supplementary Information**

**Cloning oligos**

shMYC #1

Forward CCGGCCTGAGACAGATCAGCAACAACTCGAGTTGTTGCTGATCTGTCTCAGGTTTTTG

Reverse AATTCAAAAACCTGAGACAGATCAGCAACAACTCGAGTTGTTGCTGATCTGTCTCAGG

shMYC #2

Forward CCGGCAGTTGAAACACAAACTTGAACTCGAGTTCAAGTTTGTGTTTCAACTGTTTTTG

Reverse AATTCAAAAACAGTTGAAACACAAACTTGAACTCGAGTTCAAGTTTGTGTTTCAACTG

shCIP2A #1

Forward CCGGTGCGGCACTTGGAGGTAATTTCTCGAGAAATTACCTCCAAGTGCCGCATTTTTG

Reverse AATTCAAAAATGCGGCACTTGGAGGTAATTTCTCGAGAAATTACCTCCAAGTGCCGCA

shCIP2A #2

Forward CCGGATTTGTGACTTCGTAACAATACTCGAGTATTGTTACGAAGTCACAAATTTTTTG

Reverse AATTCAAAAAATTTGTGACTTCGTAACAATACTCGAGTATTGTTACGAAGTCACAAAT

**siRNAs**

siluc Sense CGUACGGGGAAUACUUCGA

Antisense UCGAAGUAUUCCCCGUACG

siE6/E7 Sense CUUCGGUUGUGCGUACAAAGC

Antisense GCUUUGUACGCACAACCGAAG

**Antibodies**

WB = Western Blot

IF = Immunofluorescence

| **Antibody** | **Produced in** | **Manufacturer** | **Clone/ Cat. N.** | **Application** | **Dilution** |
| --- | --- | --- | --- | --- | --- |
| Vinculin | Mouse | Merck | hVIN1 | WB | 1:10 000 |
| GAPDH | Mouse | Abcam | 6C5 | WB | 1:5 000 |
| LC3 | Rabbit | Merck | L7543 | WB | 1:2 000 |
|  |  |  |  | IF | 1:200 |
| p62 | Mouse | Novus/Abnova | 2C11 | WB | 1:2 000 |
| Beclin1 | Rabbit | ProteinTech | 11306-1-AP | WB | 1:2 000 |
| c-MYC | Mouse | Calbiochem | Ab1 | WB | 1:500 |
| c-MYC | Rabbit | Cell Signaling | 9402 | WB | 1:1 000 |
| c-MYC/n-MYC | Rabbit | Cell Signaling | D3N8F | ChIP | 10µg/IP |
| TFE3 | Rabbit | Merck | HPA023881 | WB | 1:1 000 |
|  |  |  |  | ChIP | 10µg/IP |
| TFEB | Rabbit | Cell Signaling | 4240 | WB | 1:1 000 |
| TFEB | Rabbit | Cell Signaling | D207D | ChIP | 10µg/IP |
| ß-Actin | Mouse | Santa Cruz | C4 | WB | 1:2 000 |
| Akt | Rabbit | Cell Signaling |  | WB | 1:1 000 |
| p-Akt(Ser473) | Rabbit | Cell Signaling | 193H12 | WB | 1:1 000 |
| p70S6K | Rabbit | Cell Signaling | 2708 | WB | 1:1 000 |
| p-p70S6K(Thr389) | Rabbit | Cell Signaling | 92345 | WB | 1:1 000 |
| CIP2A | Rabbit | Elabscience | E-AB-14875 | WB | 1:2 000 |
| HPV16 E7 | Mouse | Santa Cruz | NM2 | WB | 1:500 |
| p53 | Mouse | Santa Cruz | DO-1 | WB | 1:1 000 |
| LAMP2 | Mouse | Santa Cruz | H4B4 | IF | 1:100 |

**RT-qPCR primers**

*HPV16 E6* Forward ATGTTTCAGGACCCACAGGA

Reverse CAGCTGGGTTTCTCTACGTGTT

*HPV16 E7* Forward CAGAGGAGGAGGATGAAATAGATGG

Reverse CACAACCGAAGCGTAGAGTCACAC

*RPLP0* Forward TTCATTGTGGGAGCAGAC

Reverse CAGCAGTTTCTCCAGAGC

*CTSA* Forward CCTGTGGTGCTTTGGCTCA

Reverse GGCGACCTCAGTGTCATTAG

*CTSB* Forward AGTGGAGAATGGCACACCCTA

Reverse AAGAAGCCATTGTCACCCCA

*TFE3* Forward GCTGCTTTCCTTGGC

Reverse ATCTGAGGGCGGTGC

*LAMP2*  Forward GAAAATGCCACTTGCCTTTATGC

Reverse AGGAAAAGCCAGGTCCGAAC

*LC3B1* Forward ACCATGCCGTCGGAGAAG

Reverse ATCGTTCTATTATCACCGGGATTTT

*GABARAP* Forward CGGGTGCCGGTGATAGTAGA

Reverse TGAGATCAGAAGGCACCAGGTA

*BECN1* Forward TGTCACCATCCAGGAACTCA

Reverse CTGTTGGCACTTTCTGTGGA

*GABARAPL1* Forward TTTGGTGCCCCTTATCTCAC

Reverse GGCCATCATGTAGCATTCCTT

*MYC* Forward TTCGGGTAGTGGAAAACCAG

Reverse CAGCAGCTCGAATTTCTTCC

*E2F1* Forward TGCAGAGCAGATGGTTATGG

Reverse ATCTGTGGTGAGGGATGAGG

*TFEB* Forward CCAGAAGCGAGAGCTCACAGAT

Reverse TGTGATTGTCTTTCTTCTGCCG

*LAMP1*  Forward ACGTTACAGCGTCCAGCTCAT

Reverse TCTTTGGAGCTCGCATTGG

*SQSTM1* Forward AGGCGCACTACCGCGAT

Reverse CGTCACTGGAAAAGGCAACC

*CIP2A* Forward GCCACACTGATTCGGTGTTTT

Reverse TGCCGACAAAGATTTGCCAATA

*CTSD* Forward AACTGCTGGACATCGCTTGCT

Reverse CATTCTTCACGTAGGTGCTGGA

*ATP6V0E1* Forward CATTGTGATGAGCGTGTTCTG

Reverse AACTCCCCCGGTTAGGACCCTT

**ChIP-qPCR primers**

All primers are designed on the promoter region of the gene within the TF binding region.

*GLA* Forward TAGCGAGACGGTAGACGAC

Reverse ACCCGCCCTATTTCCATAC

*MCOLN1* Forward AGGGGCTCTGGGCTACC

Reverse GCCCGCCGCTGTCACTG

*MAP1LC3B* Forward TCTTACAGCCACCAGGAGAGTT

Reverse TTTGTCCCGAGCCTTCATTCTG

*SQSTM1* Forward AACTGGGAACTTCTCTGGTGCT

Reverse AAGCTGGCTCTCAGGTTGCTGTT
