# Supplementary material for: c-MYC-dependent transcriptional inhibition of autophagy is implicated in cisplatin sensitivity in HPV-positive head and neck cancer
Source: Cell Death Dis. 2023 Nov 4;14(11):719. doi: 10.1038/s41419-023-06248-3 (PMC10625625; doi:10.1038/s41419-023-06248-3)

ORIGINAL BLOTS


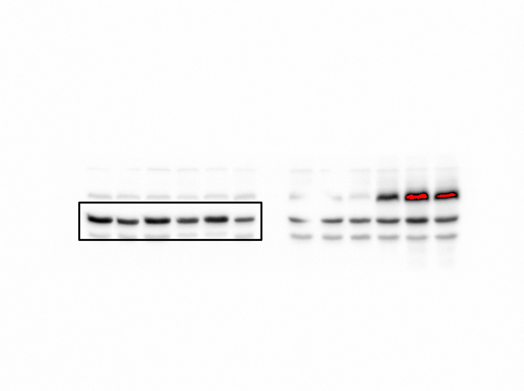


**Figure 1C**

- Actin


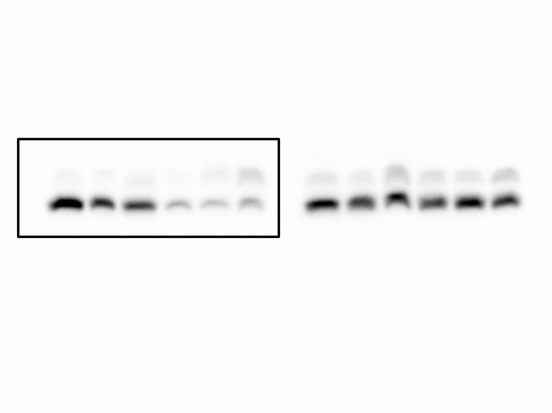


- LC3 I-II

**
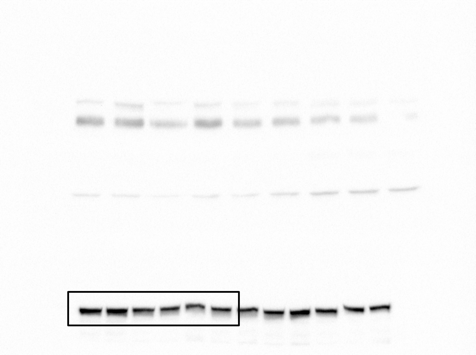
Figure 2C**

- Vinculin
-
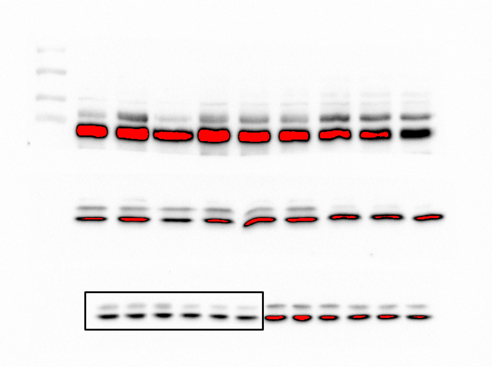
LC3 I-II

**
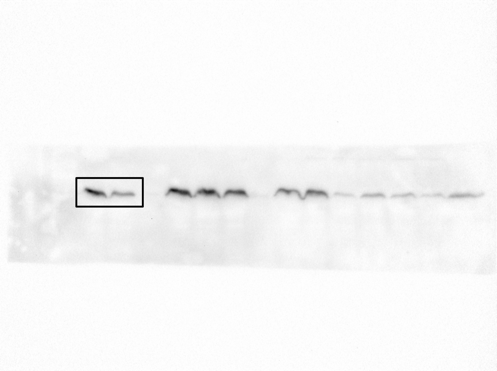
Figure 2F**

- P53


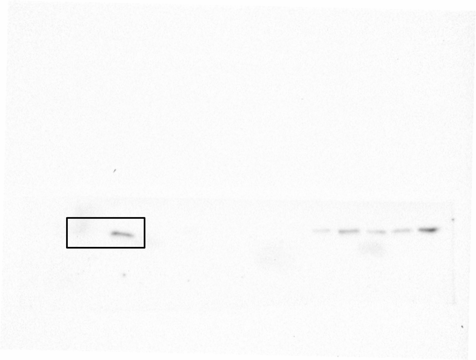


- HPV16 E7


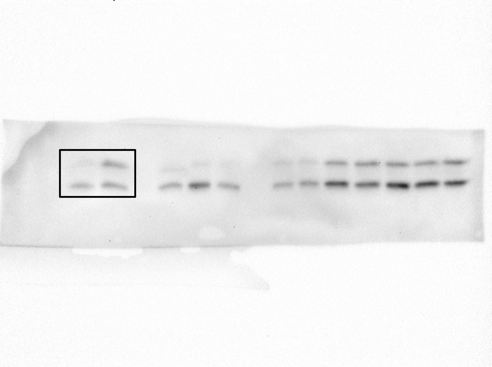


- LC3 I-II


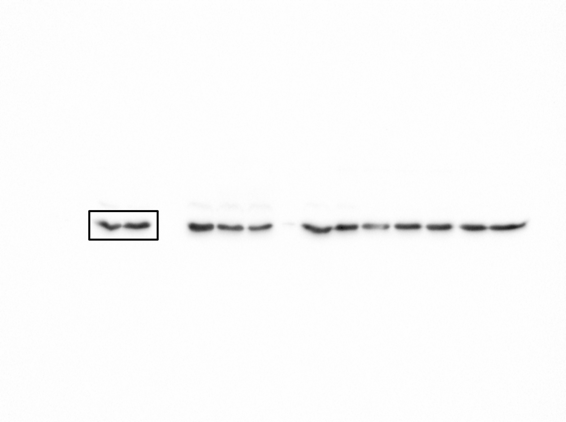


- Actin

**
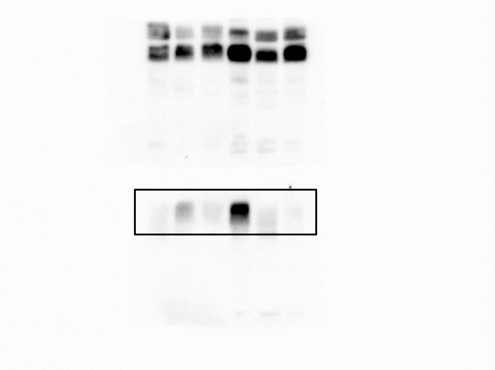
Figure 3A**

- TFEB


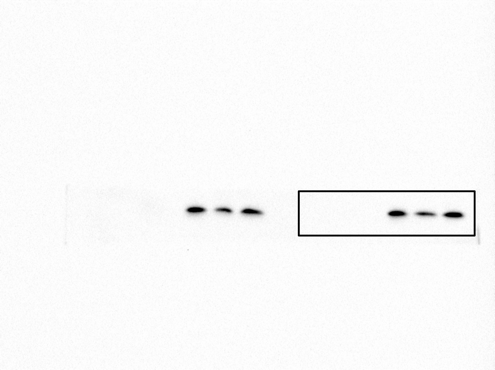


- HPV16 E7
- TFE3


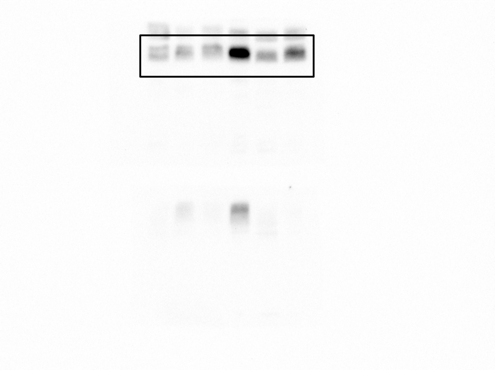


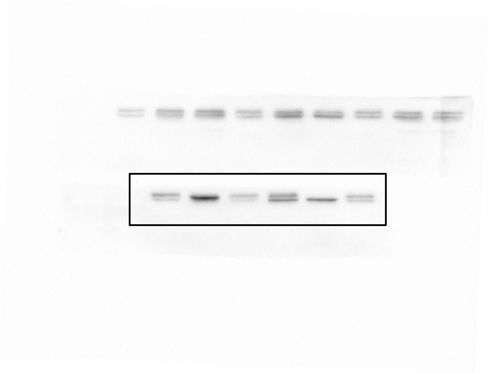


- c-MYC


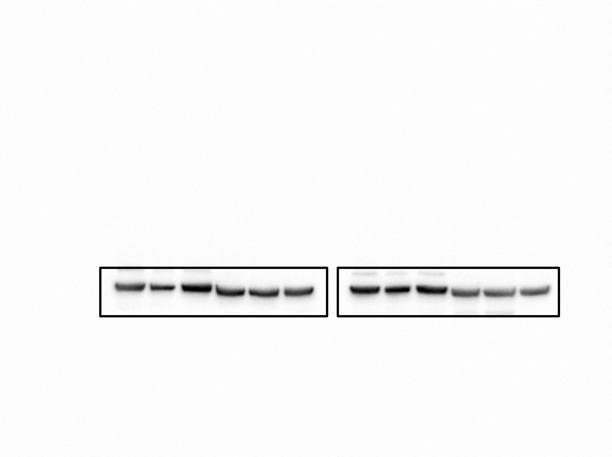


-
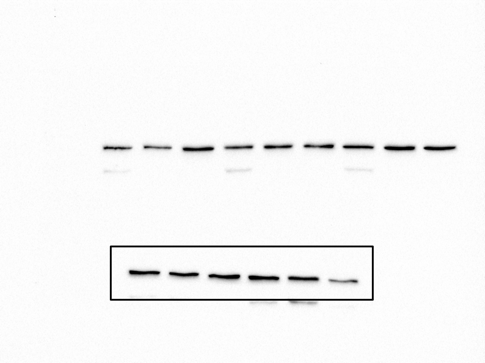
Vinculins

**
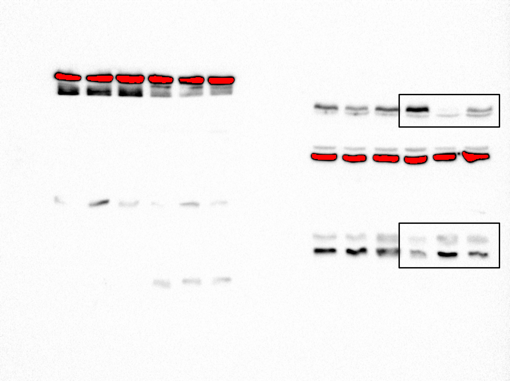
Figure 4A**

- c-MYC and LC3 I-II


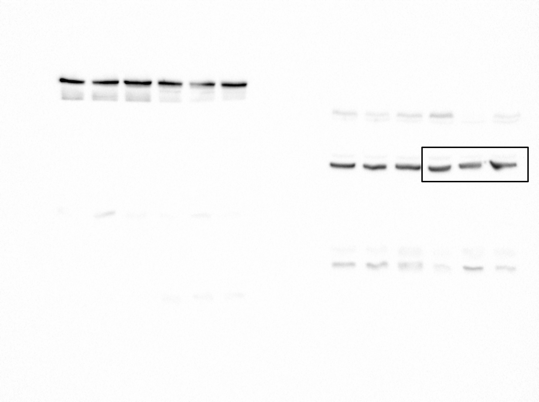


- GAPDH


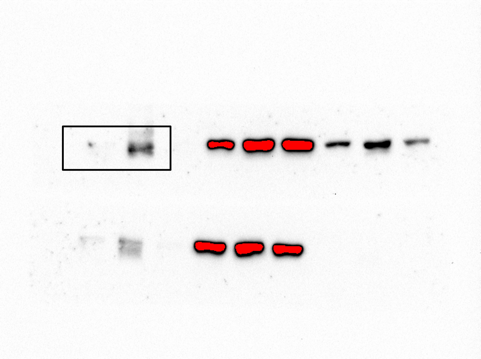
**Figure 4C**

- c-MYC


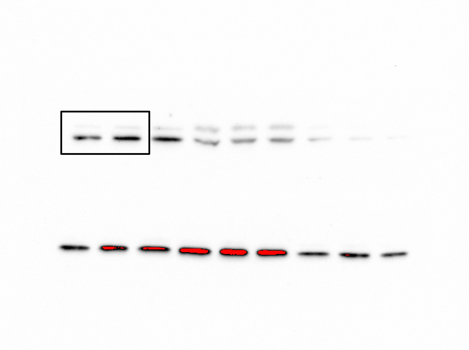


- LC3 I-II


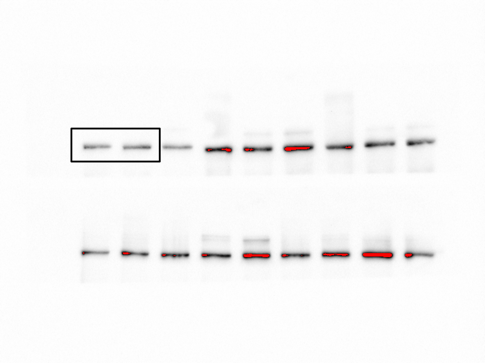


- Vinculin


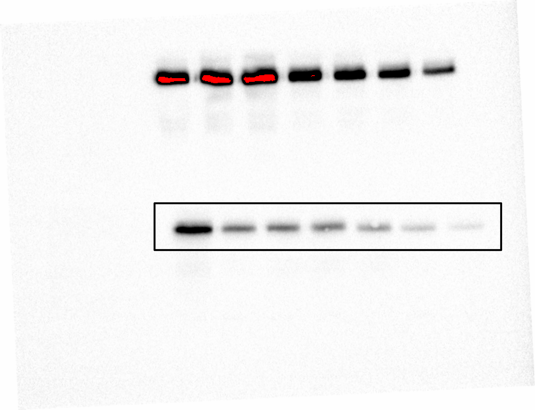

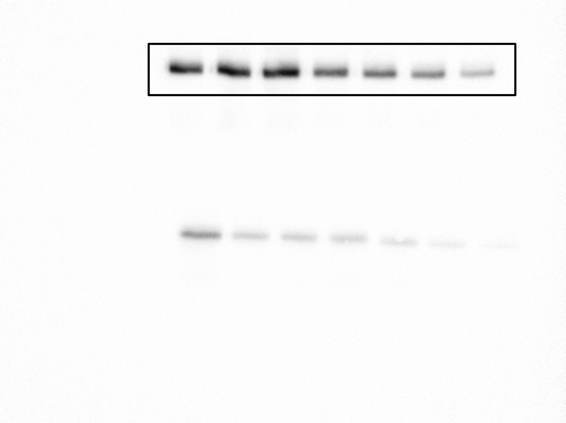
**Figure 4E**

- c-MYC


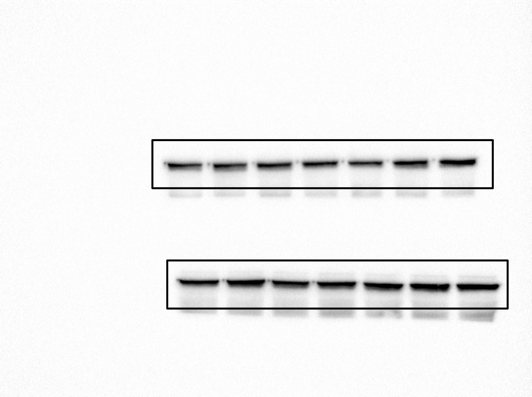


- Vinculin


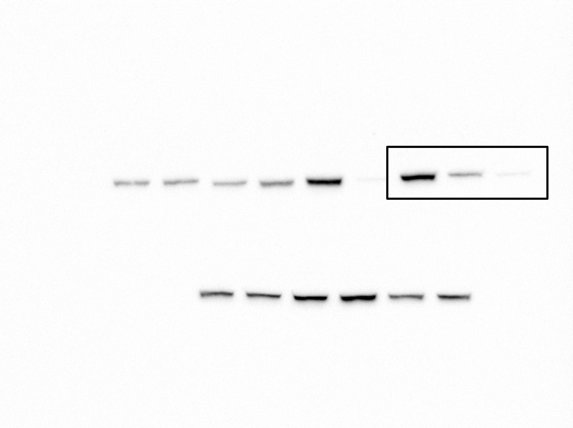
**Figure 4H**

- CIP2A


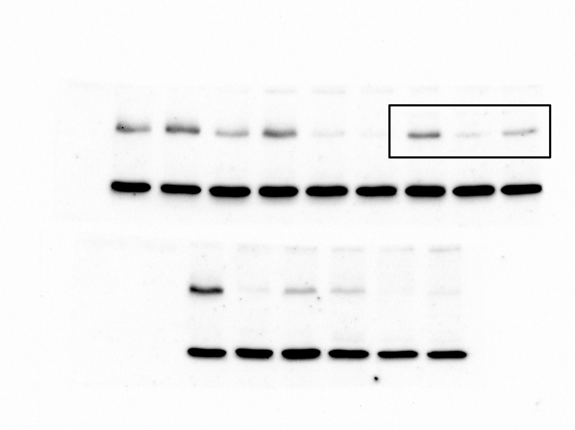


- c-MYC


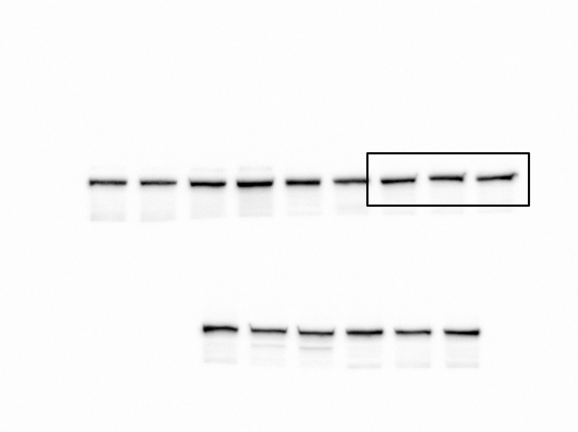


- Vinculin


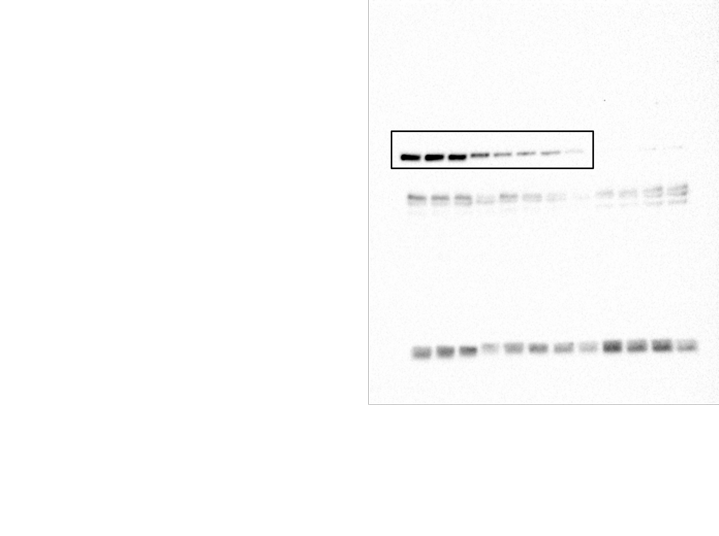
**Figure 4I**

- CIP2A
-
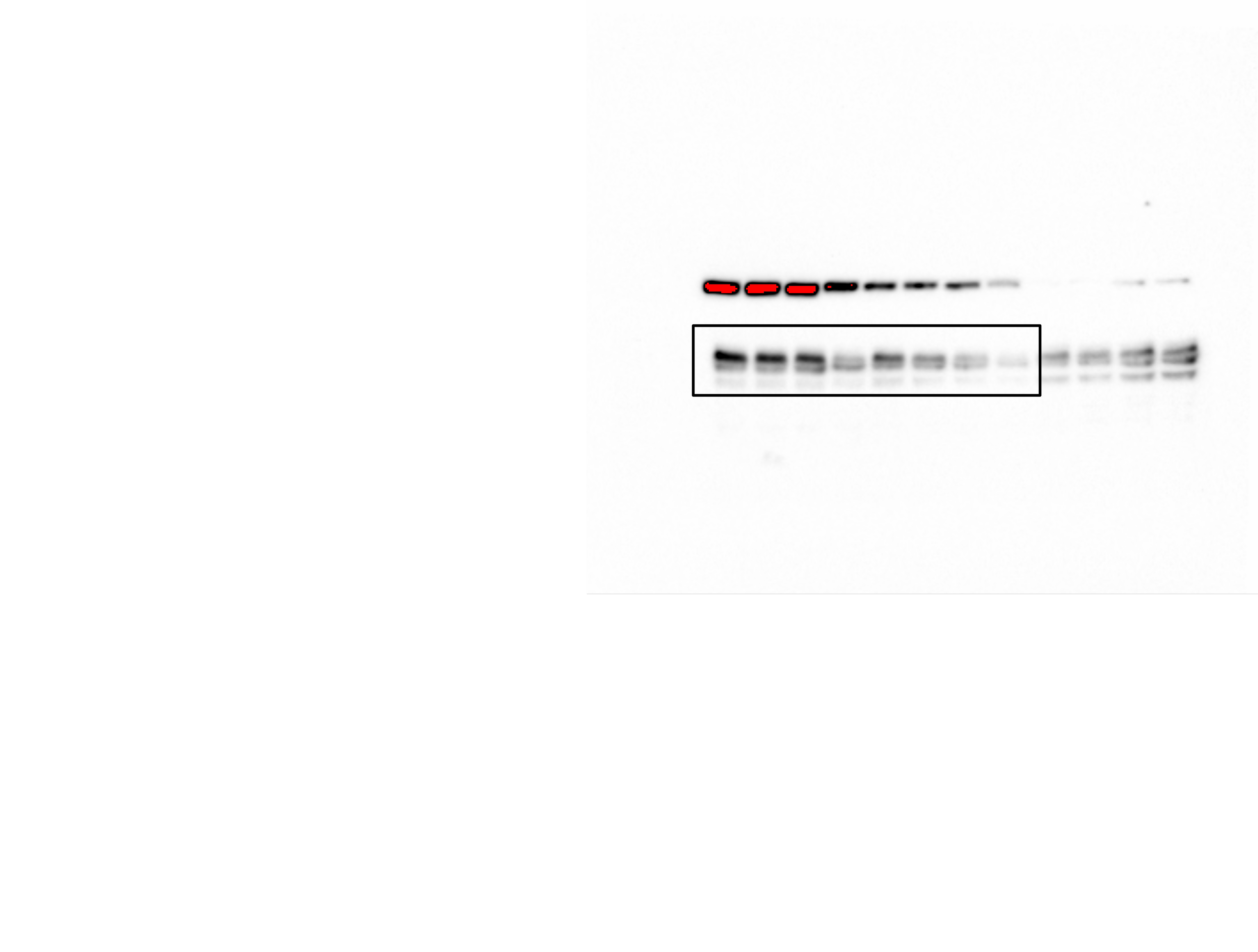
c-MYC


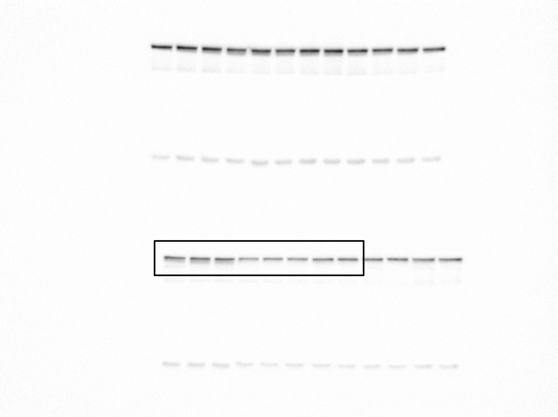


- Vinculin


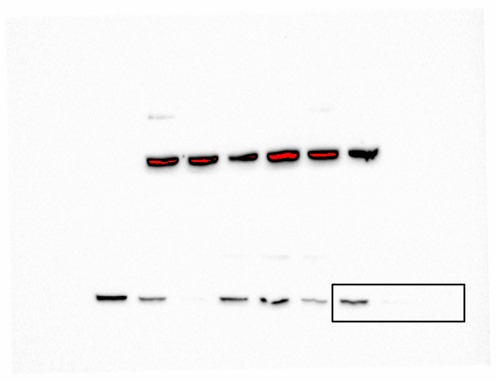
**Figure 5E**

- CIP2A


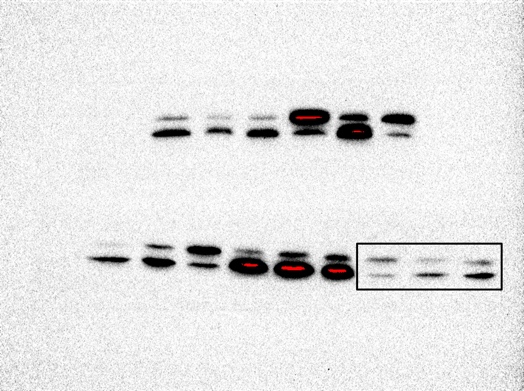


- LC3 I-II
-
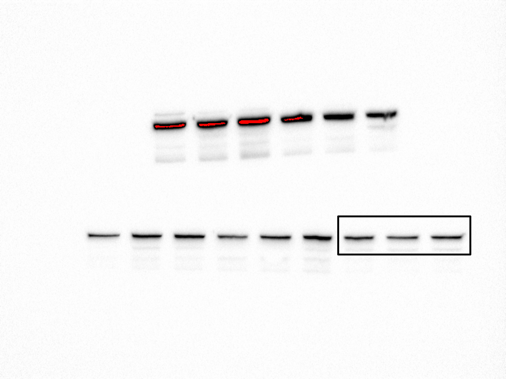
Vinculin


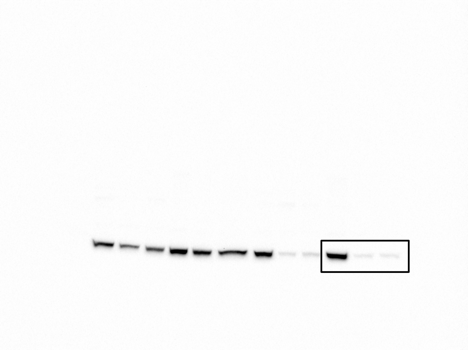
**Figure 5F**

- CIP2A


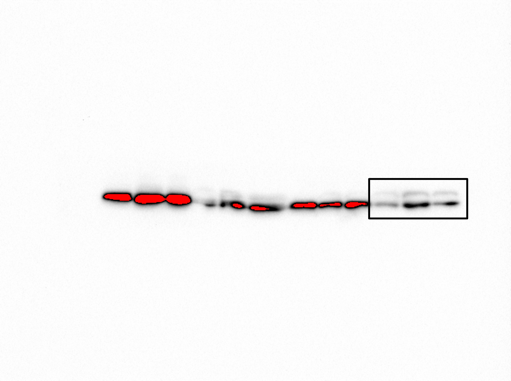


- LC3 I-II


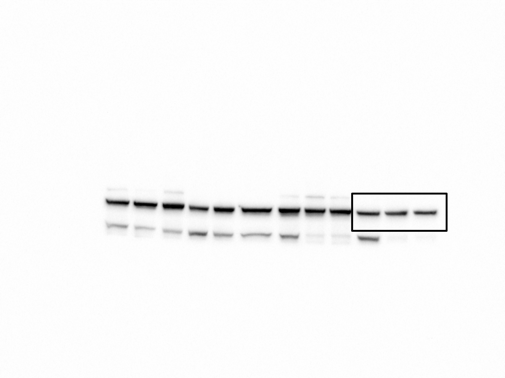


- Vinculin


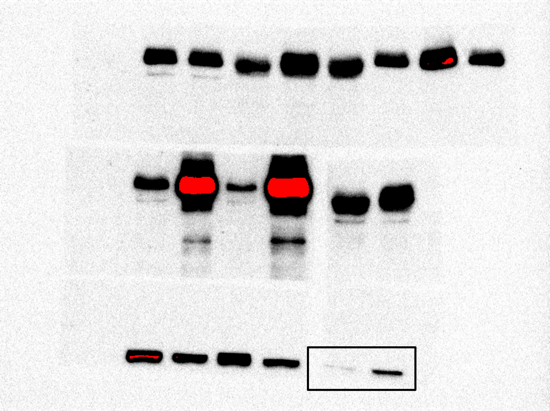


**Figure 6D**

- CIP2A
- p53


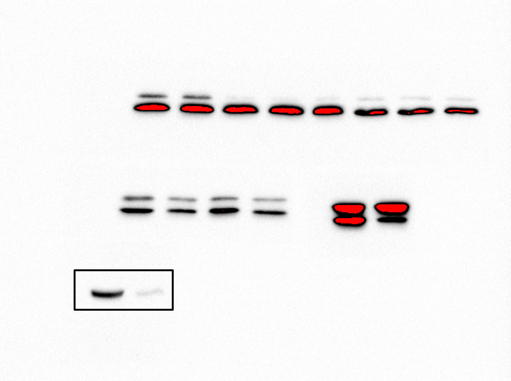


-
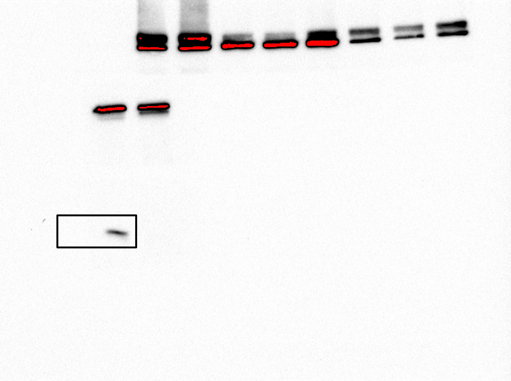
HPV16 E7
-
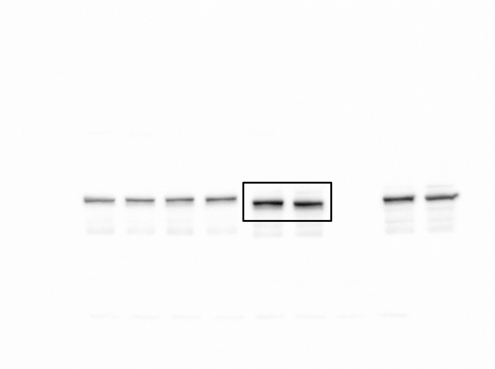
Vinculin

**Figure 6E**

- CIP2A


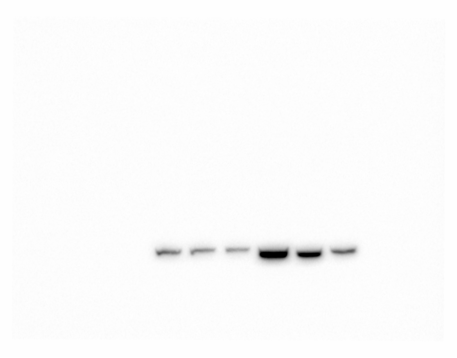


- HPV16 E7


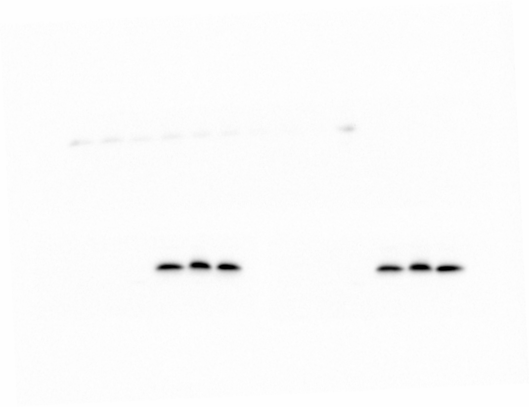


-
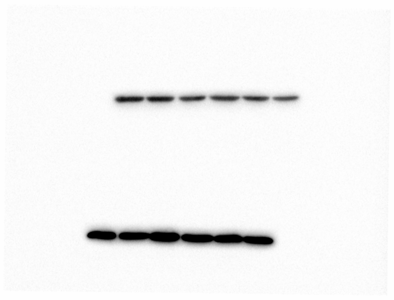
Actin


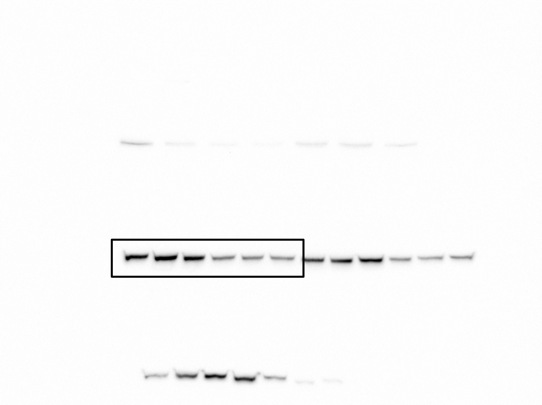
**Figure 6G**

- CIP2A


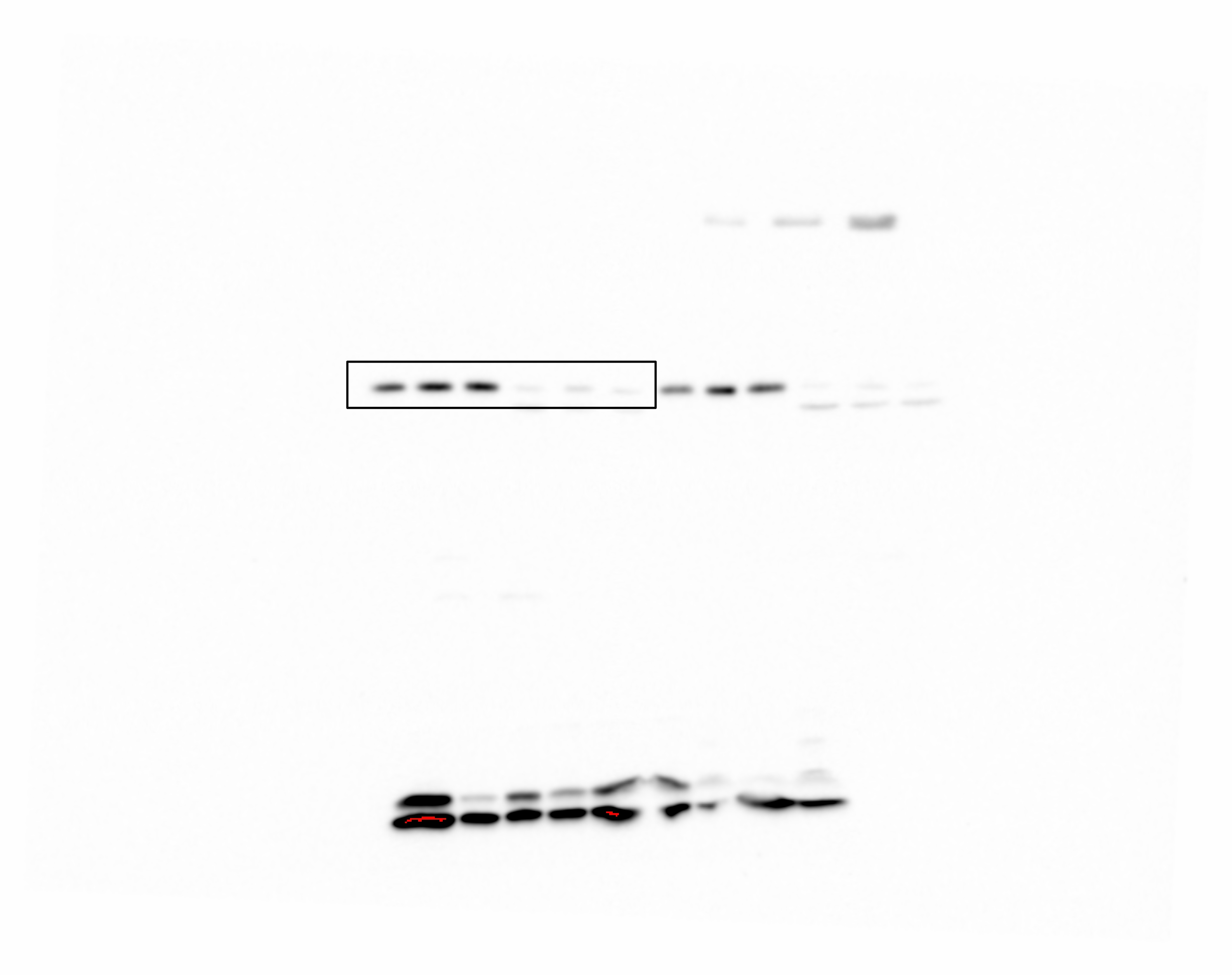


- HPV16 E7
- Vinculin


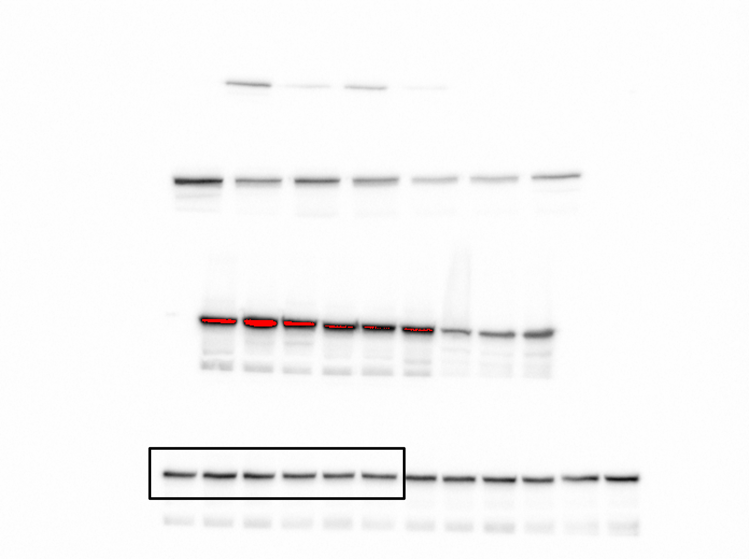


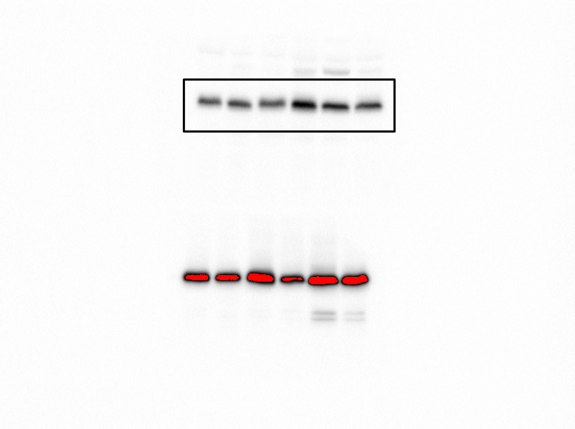


**Figures S1B-S1C**

- Beclin
-
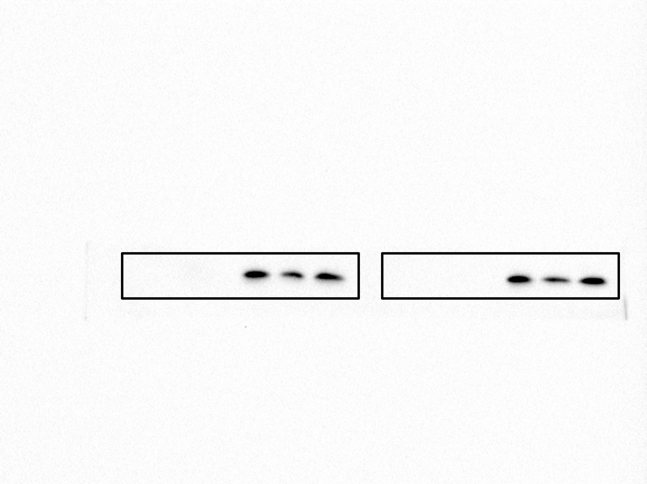
HPV16 E7
-
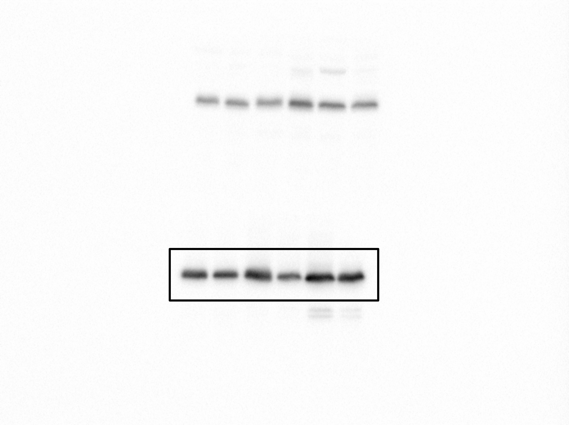
p62


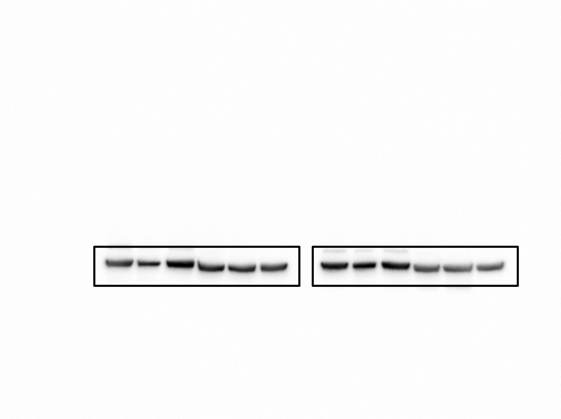


- Vinculin

**
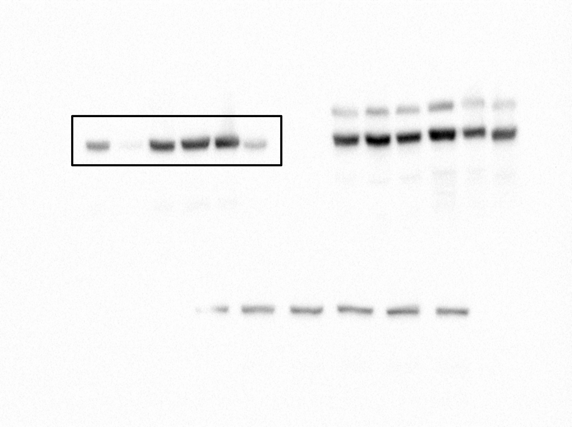
Figures S1D-S1E**

- p-AktS473


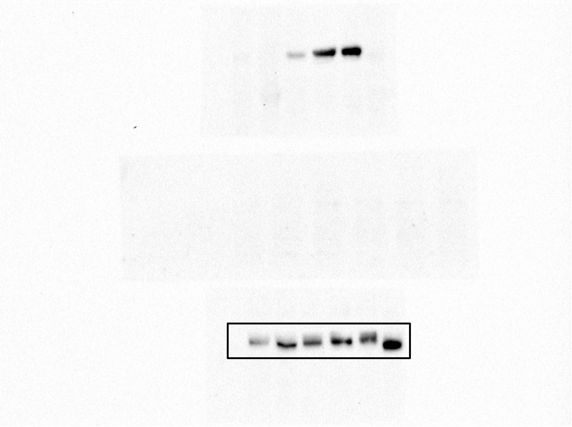


- Akt
-
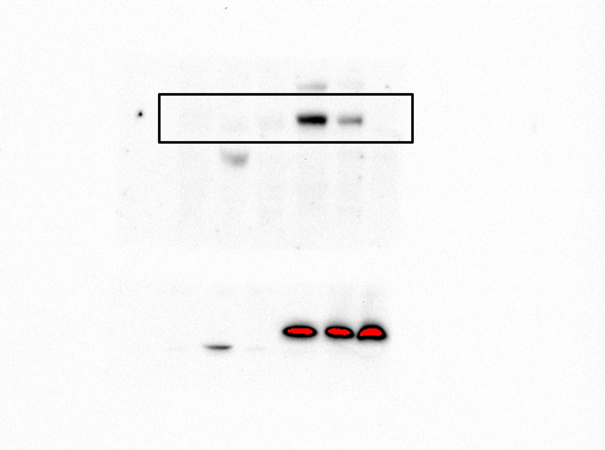
p-p70S6K


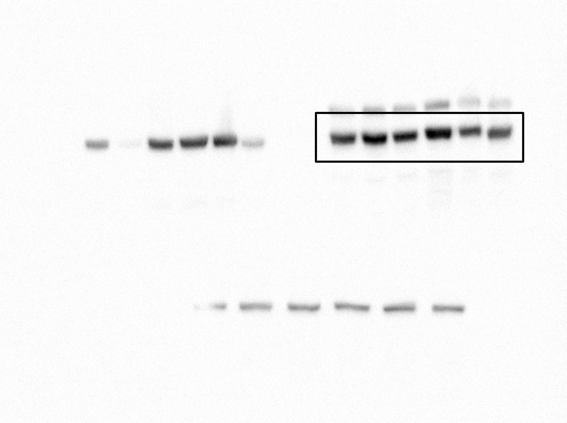


- p70S6K


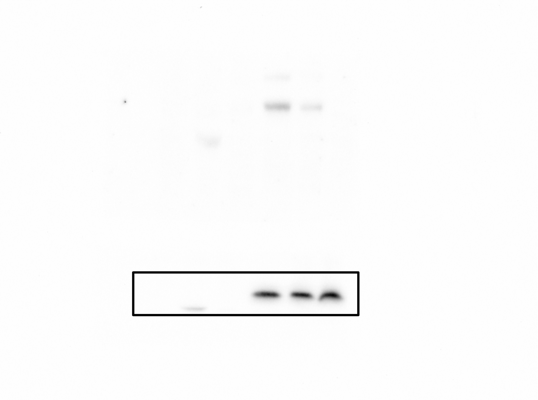


- HPV16 E7


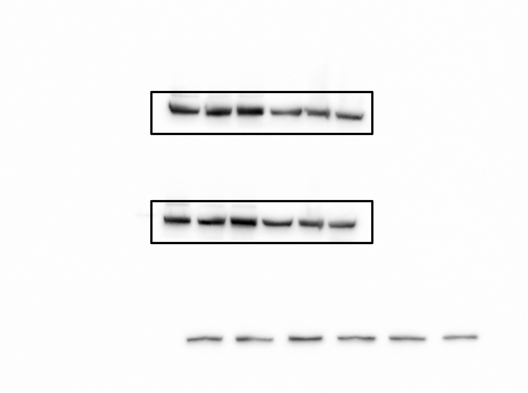


- Vinculin


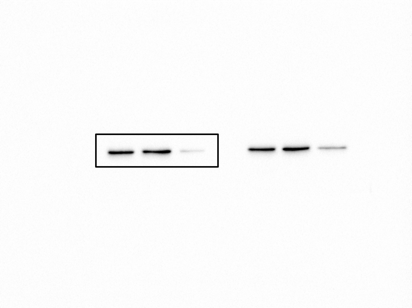
**Figure S2A**

- p53
-
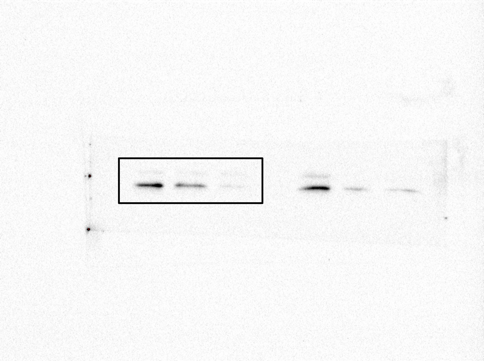
LC3 I-II


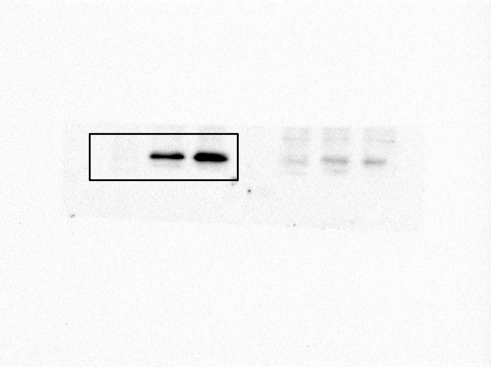


- HPV16 E7


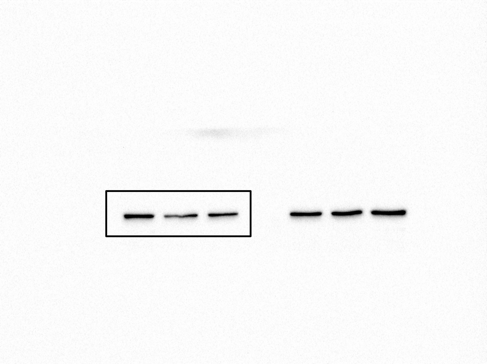


- Vinculin


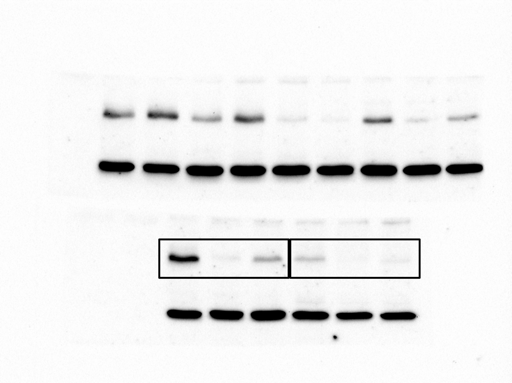
**Figure S4A-S4B**

- c-MYC


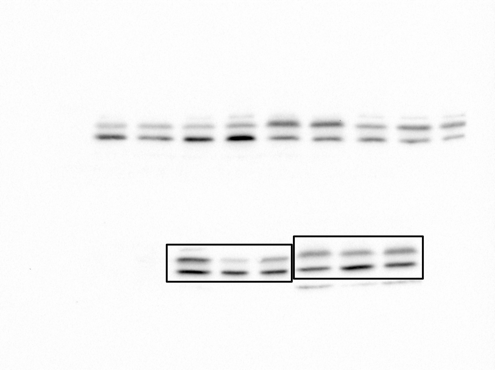


- LC3 I-II
-
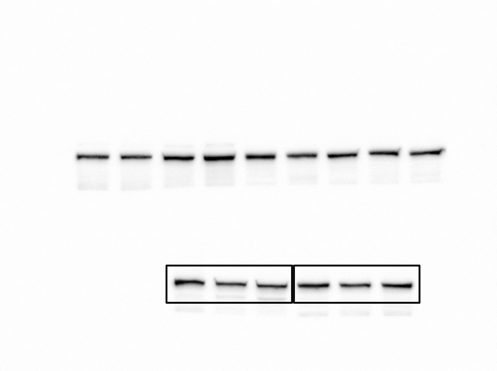
Vinculin


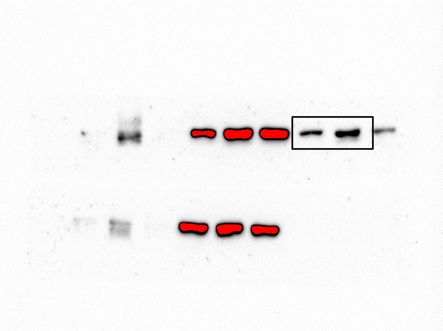
**Figure S4C**

- c-MYC


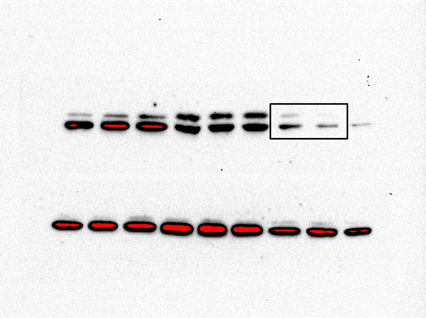


- LC3 I-II


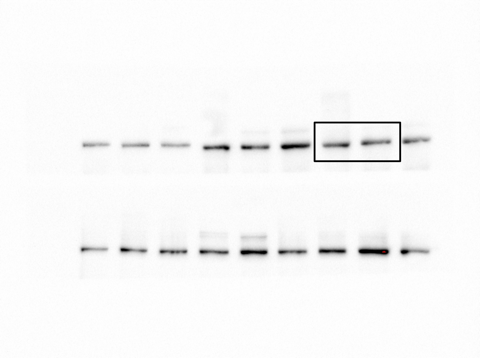


- Vinculin

**Figure S4E**

- c-MYC


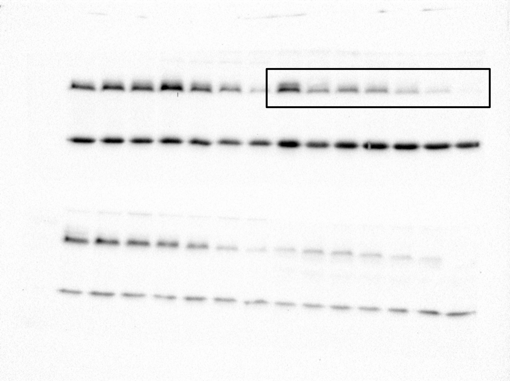

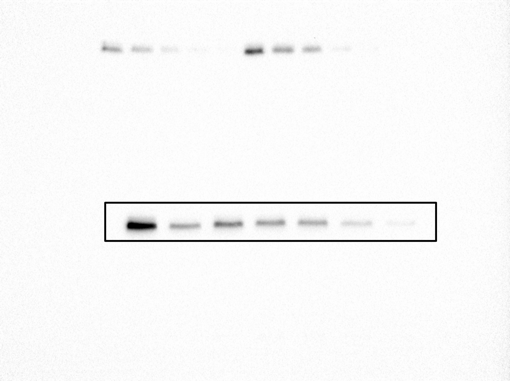


-
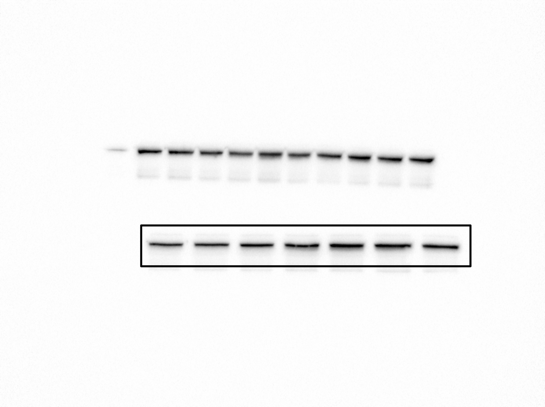
Vinculin
-
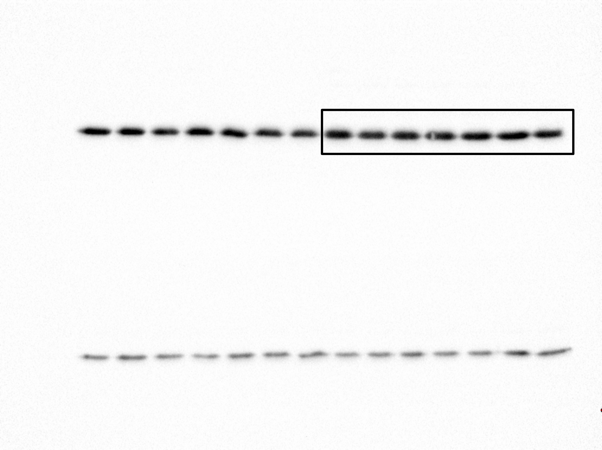
GAPDH

**Figure S4F**


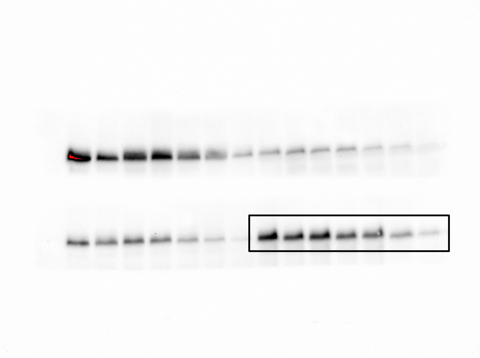

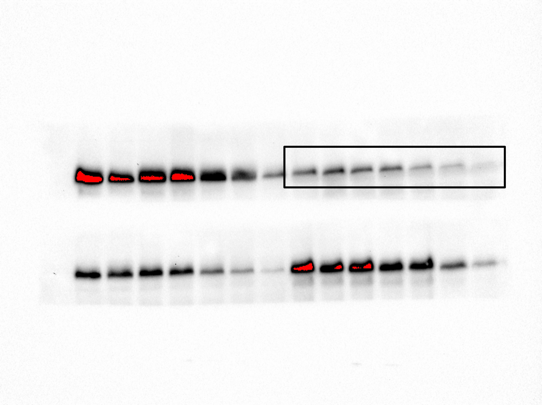


- c-MYC


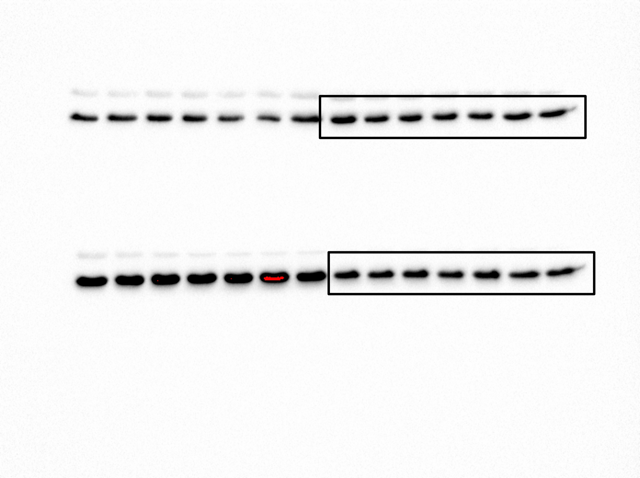


- GAPDH

**Figure S7C**

- LC3 I-II


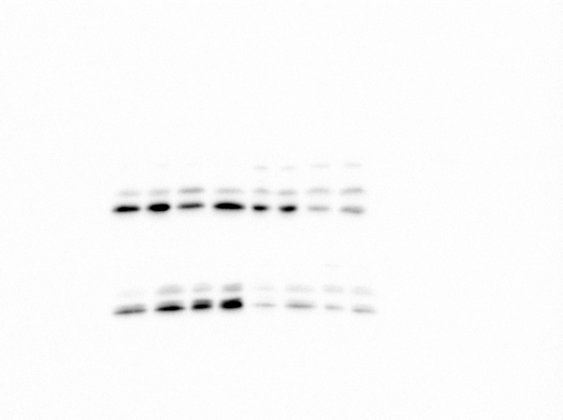


- Vinculin


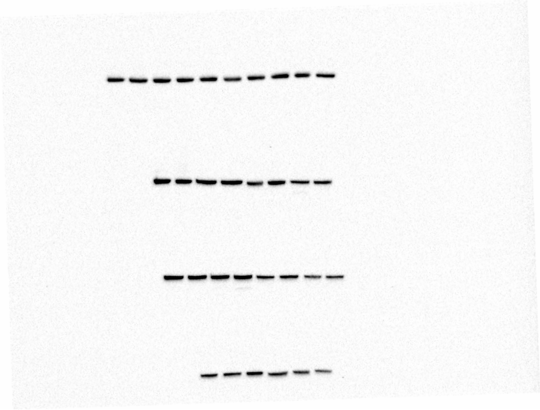


**Figure S7D-E**

- LC3 I-II


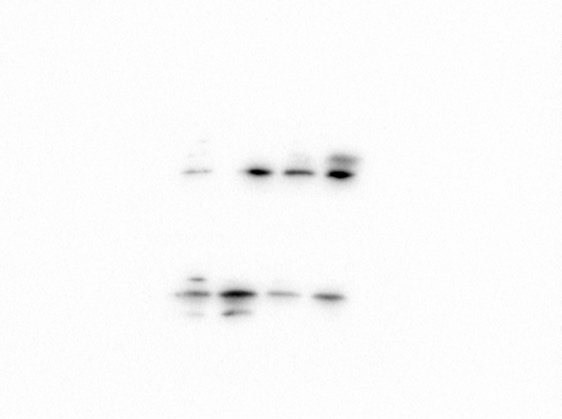


- Actin


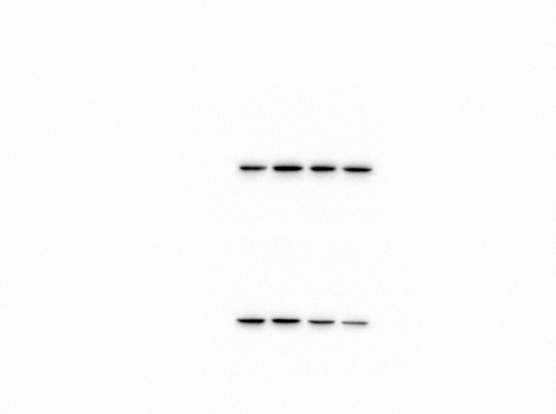

Supplement: Supplementary file 11 — Original Blots [file 41419_2023_6248_MOESM11_ESM.docx]
